# A cross-talk between epithelium and endothelium mediates human alveolar–capillary injury during SARS-CoV-2 infection

**DOI:** 10.1038/s41419-020-03252-9

**Published:** 2020-12-08

**Authors:** Peng Wang, Ronghua Luo, Min Zhang, Yaqing Wang, Tianzhang Song, Tingting Tao, Zhongyu Li, Lin Jin, Hongyi Zheng, Wenwen Chen, Mengqian Zhao, Yongtang Zheng, Jianhua Qin

**Affiliations:** 1 Division of Biotechnology, CAS Key Laboratory of SSAC, Dalian Institute of Chemical Physics, Chinese Academy of Sciences, Dalian, China; 2 Kunming National High-level Bio-safety Research Center for Non-human Primates, Center for Biosafety Mega-Science, Kunming Institute of Zoology, Chinese Academy of Sciences, Kunming, 650107 China; 3 Key Laboratory of Animal Models and Human Disease Mechanisms of Chinese Academy of Sciences, KIZ-CUHK Joint Laboratory of Bioresources and Molecular Research in Common Diseases, Kunming Institute of Zoology, Chinese Academy of Sciences, Kunming, Yunnan 650223 China; 4 University of Chinese Academy of Sciences, Beijing, China; 5 Key Laboratory of Animal Models and Human Disease Mechanisms of Chinese Academy of Sciences, Kunming Institute of Zoology, Chinese Academy of Sciences, Kunming, 650223 Yunnan China; 6 CAS Center for Excellence in Brain Science and Intelligence Technology, Chinese Academy of Sciences, Shanghai, China; 7 Institute for Stem Cell and Regeneration, Chinese Academy of Sciences, Beijing, China

**Keywords:** Mechanisms of disease, Viral infection

## Abstract

COVID-19, caused by SARS-CoV-2, is an acute and rapidly developing pandemic, which leads to a global health crisis. SARS-CoV-2 primarily attacks human alveoli and causes severe lung infection and damage. To better understand the molecular basis of this disease, we sought to characterize the responses of alveolar epithelium and its adjacent microvascular endothelium to viral infection under a co-culture system. SARS-CoV-2 infection caused massive virus replication and dramatic organelles remodeling in alveolar epithelial cells, alone. While, viral infection affected endothelial cells in an indirect manner, which was mediated by infected alveolar epithelium. Proteomics analysis and TEM examinations showed viral infection caused global proteomic modulations and marked ultrastructural changes in both epithelial cells and endothelial cells under the co-culture system. In particular, viral infection elicited global protein changes and structural reorganizations across many sub-cellular compartments in epithelial cells. Among the affected organelles, mitochondrion seems to be a primary target organelle. Besides, according to EM and proteomic results, we identified Daurisoline, a potent autophagy inhibitor, could inhibit virus replication effectively in host cells. Collectively, our study revealed an unrecognized cross-talk between epithelium and endothelium, which contributed to alveolar–capillary injury during SARS-CoV-2 infection. These new findings will expand our understanding of COVID-19 and may also be helpful for targeted drug development.

## Introduction

Since outbreak at the end of 2019, the COVID-19 pneumonia epidemic spreads rapidly around the world and causes a serious global social crisis. As of early October, SARS-CoV-2 has infected over 33 million individuals and caused more than 1 million deaths (World Health Organization, 2020). Clinically, SARS-CoV-2 primarily infects human lung and causes severe injury and inflammatory changes. Within the intra-alveolar spaces of COVID-19 deaths, pneumocytes desquamation, proteinaceous exudate, interstitial inflammation, and lymphocytes infiltration is frequently detected^1–4^. Alveolus is the functional unit of lung, which plays vital roles in gas exchange and prevention of pathogen invasion^5–7^. It is mainly composed of epithelial layer and extracellular matrix surrounded by capillaries. The alveolar epithelium, its adjacent pulmonary microvascular endothelium, and extracellular matrix between them can form an alveolar–capillary barrier, serving as a host barrier. Though SARS-CoV-2 infection is localized primarily to the respiratory system, there is an increasing evidence of systemic dissemination of the virus to multiple organs, such as gastrointestinal tract, peripheral blood, urinary system, and so on^8–10^. All these clinical and pathological clues suggest the function of alveolar–capillary barrier is compromised in COVID-19, leading to the leakage of virus to the circulatory system.

Physically, the close anatomical association between alveolar epithelium and pulmonary microvascular endothelium, together with the putative distribution of viral host receptor on endothelial cell surface^11,12^, makes it important for investigation of alveolar epithelium and pulmonary microvascular endothelium in parallel following SARS-CoV-2 infection. It has been demonstrated alveolar epithelial cell (especially the alveolar epithelial type II cell; AT2) is the primary infected target; however, little is known about how pulmonary microvascular endothelium is disrupted during SARS-CoV-2 infection, let alone the epithelium-endothelium interplay in the pathogenesis of COVID-19.

Virus itself is not an independent living entity, and it relies heavily on host machineries to achieve its replication and spread^13,14^. Therefore, in order to develop targeted therapies to inhibit the viral replication and infection more effectively, it is critical to understand how the virus usurps the host cells at the molecular level. Currently, scientists have conducted a lot of works to elucidate SARS-CoV-2 virus–host interaction mechanisms by means of proteomics and bioinformatics, such as drawing SARS-CoV-2 protein interaction map in host cells, developing network based-approach on HCoV-host interactome, and building a computational repository of SARS-CoV-2 virus-host interaction mechanisms^15–17^. In this study, we aimed to explore the host responses of alveolar epithelium and pulmonary microvascular endothelium to SARS-CoV-2 infection at the molecular level in a co-culture system, together with combined proteomics techniques and transmission electron microscopy (TEM) analysis.

Our study showed that SARS-CoV-2 primarily invaded alveolar epithelial cells, and usurped their cellular machineries for viral massive replication. During the process, infected epithelial cells underwent global proteome modulations and structural remodeling across many sub-cellular compartments. Among the affected organelles, mitochondria seemed to be a primary target for virus. SARS-CoV-2 infection could activate antiviral and immune responses in epithelial cells and up-regulate proinflammatory cytokines, such as IL1α and interferons (IFNs). These cytokines may be released to extracellular space, and further induced damage (immune responses) of adjacent microvascular endothelial cells. In addition, based on proteomic analysis and EM data, we found autophagy pathway was modulated in SARS-CoV-2-infected cells. We tested some autophagy inhibitors, and identified Daurisoline had an obvious antiviral effect on SARS-CoV-2 in host cells.

Our study revealed an unrecognized epithelium-endothelium cross-talk during SARS-CoV-2 infeciton. This epithelium-endothelium cross-talk mediated pulmonary capillary injury, and may further exacerbate the disease.

## Results

### Alveolar epithelial cells were more susceptible to SARS-CoV-2 infection than pulmonary microvascular endothelial cells

**Fig. 1:**
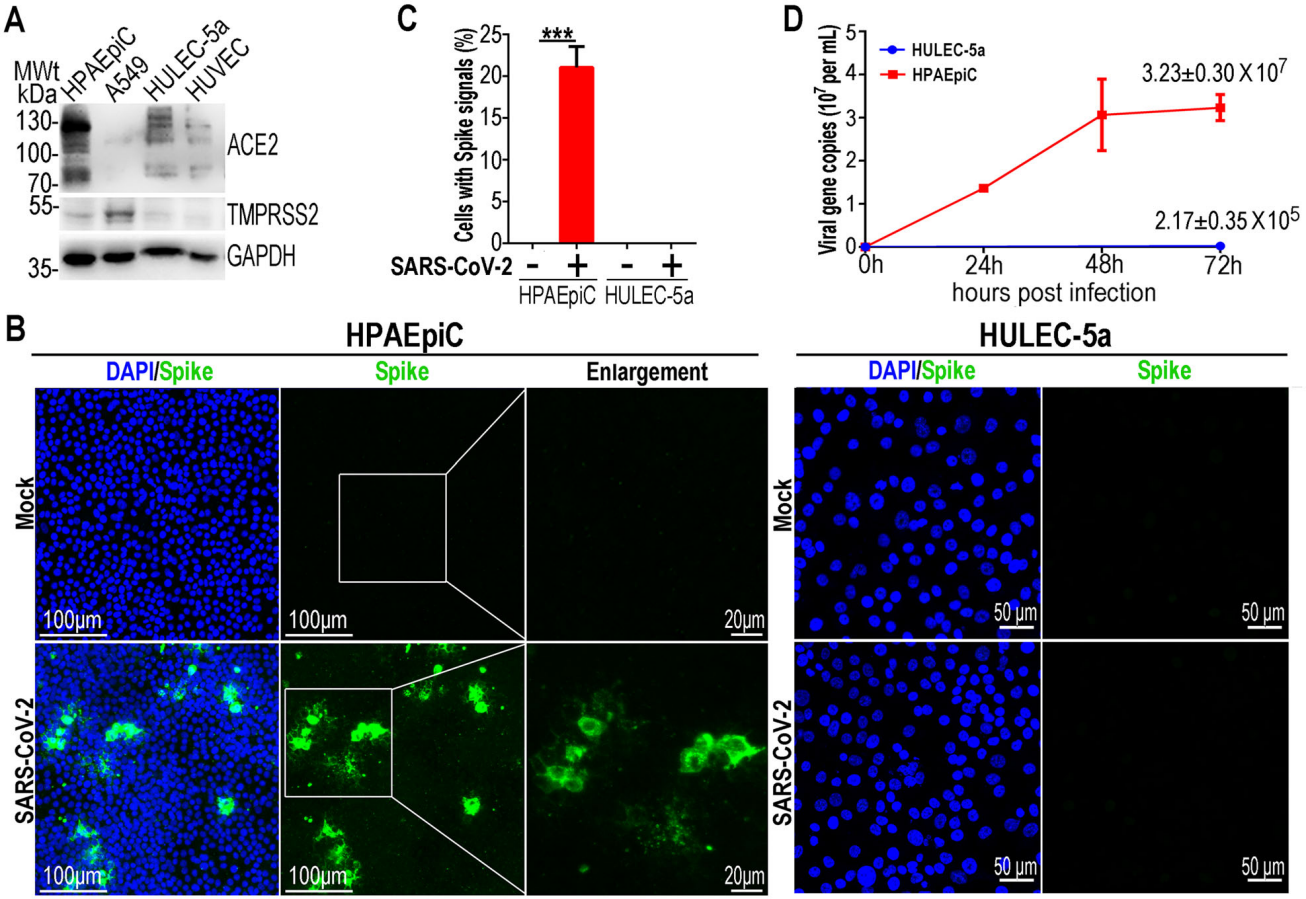
HPAEpiC cells were more susceptible to SARS-CoV-2 infection than HULEC-5a cells. **a** Western blot was performed to analyze the expression level of ACE2 and TMPRSS2 in four cell lines. The result was a representative blot from three independent experiments. GAPDH was served as a loading control. **b** Confocal fluorescent images of cells immunostained for viral Spike protein (viral Spike protein S1 subunit) 72 h post-infection. **c** Quantification of the percentage of HPAEpiC cells or HULEC-5a cells immunostained by viral Spike protein. Data represent four independent experiments, and more than 1000 cells for each group were quantified. Data were presented as mean ± SEM. Data were analyzed by Student’s *t* test (***: *P* < 0.001). **d** Culture supernatants of HPAEpiC cells or HULEC-5a cells were harvested at indicated time points following SARS-CoV-2 infection to examine the viral load using qRT-PCR for different groups. The average of two independent experiments was shown. Data were presented as mean ± SEM.

It is well known that SARS-CoV-2 utilizes ACE2 as a receptor for cellular entry and the serine protease TMPRSS2 for viral Spike protein priming^18,19^. Prior to SARS-CoV-2 infection, two alveolar epithelial cell lines and two vascular endothelial cell lines were analyzed by western blot for these two proteins. Among the cells, HPAEpiC is a human AT2 cell-derived cell line^20^, and HULEC-5a is a human pulmonary microvascular endothelial cell line^21^. Western blot showed HPAEpiC cells expressed ACE2 protein at a higher level (Fig. 1a). This is consistent with scRNA-seq analysis that ACE2 is specifically expressed in AT2 cells within human lung^11,22,23^. At the meanwhile, considering HULEC-5a cell is a lung-related microvascular endothelial cell line, so we chose HPAEpiC cells and HULEC-5a cells for the following experiments.

To determine the respective susceptibility to viral infection, HPAEpiC cells and HULEC-5a cells were inoculated with SARS-CoV-2 virus at an MOI (multiplicity of infection) of 10, separately. 72 h post-infection, viral Spike protein expression can be detected in 21.06 ± 2.50% of HPAEpiC cells (Fig. 1b, c); while in HULEC-5a cells, viral Spike expression was hardly detected by immunofluorescent analysis. Furthermore, qRT-PCR was performed to examine the viral load in culture supernatants of HPAEpiC cells and HULEC-5a cells, and the results showed SARS-CoV-2 replicated much more rapidly in HPAEpiC cells than HULEC-5a cells (3.23 ± 0.30 × 10^7^ copies/mL in HPAEpiC cells Vs 2.17 ± 0.35 × 10^5^ copies/mL in HULEC-5a cells 72 h post-infection) (Fig. 1d).

### SARS-CoV-2 infection affected alveolar epithelial cells and pulmonary microvascular endothelial cells in different manners

Generally, virus targets host organelles after cellular entry, and usurps their functions for viral replication. We then examined how SARS-CoV-2 affects host cells at the organelle level by confocal microscope. HPAEpiC cells and HULEC-5a cells were infected by SARS-CoV-2 at an MOI of 10 separately, and imaged at 72 h post-infection. Organelle-specific antibodies were used to label different organelles, including endoplasmic reticulum (PDI), Golgi apparatus (GORASP2), mitochondria (ATP5A1) and peroxisomes (PEX14).

**Fig. 2:**
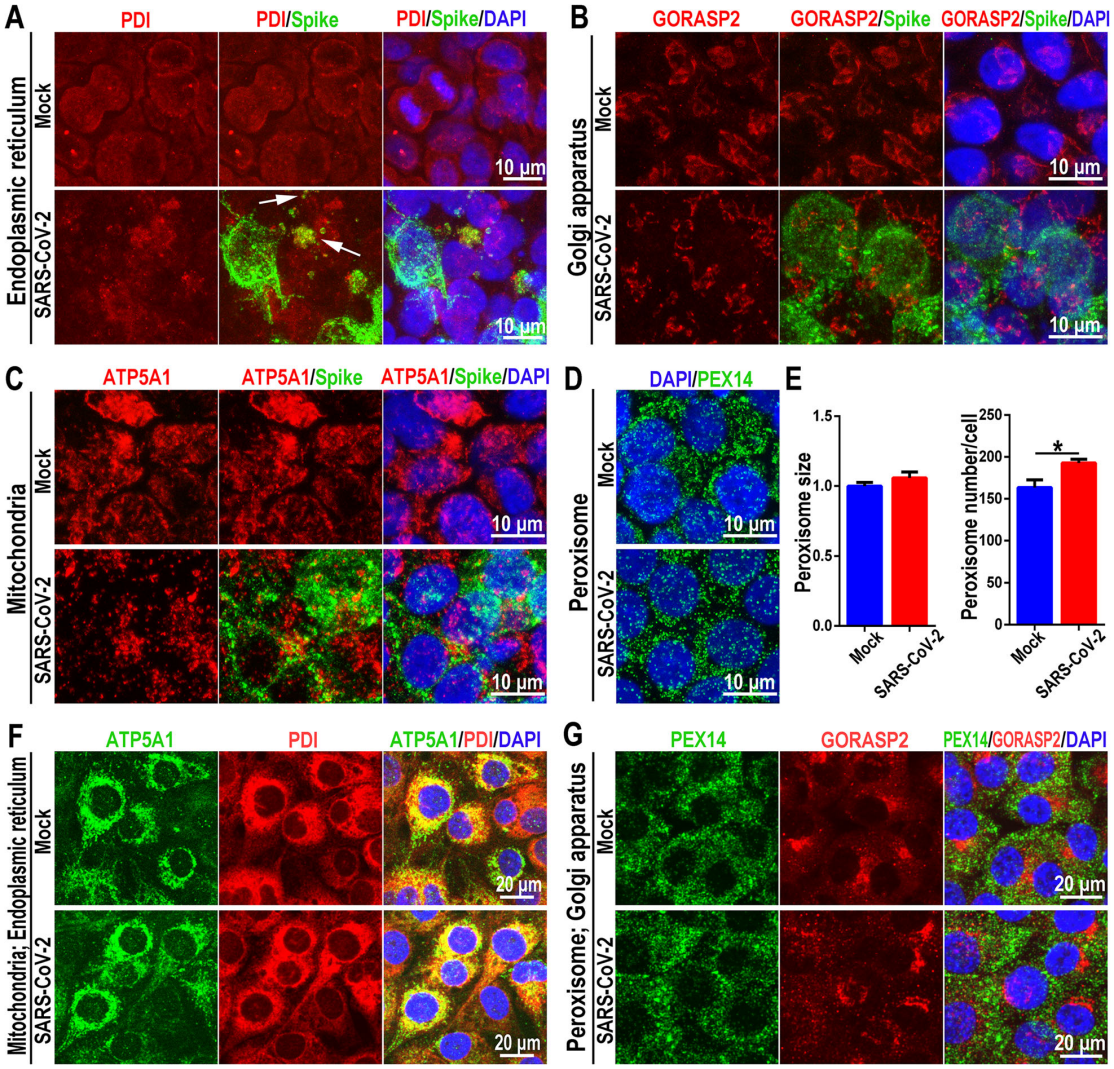
Organelles of HPAEpiC cells and HULEC-5a cells following SARS-CoV-2 infection. **a** Representative images of endoplasmic reticulum immunostained with anti-PDI antibody for mock- or SARS-CoV-2-infected HPAEpiC cells. The white arrows indicated the co-localization of virus particles and endoplasmic reticulum within the host cells. **b** Representative images of Golgi apparatus immunostained with anti-GORASP2 antibody for mock- or SARS-CoV-2-infected HPAEpiC cells. **c** Representative images of mitochondria immunostained with anti-ATP5A1 antibody for mock- or SARS-CoV-2-infected HPAEpiC cells. **d** Representative images of peroxisomes immunostained with anti-PEX14 antibody for mock- or SARS-CoV-2-infected HPAEpiC cells. **e** Quantification of peroxisome size and density (number/cell) for mock- or infected cells in (**d**). Data were analyzed by Student’s *t* test (*: *P* < 0.05). Data represent four independent experiments, and more than 150 cells for each group were quantified. Data were presented as mean ± SEM. **f** Representative images of mitochondria and endoplasmic reticulum of mock- or SARS-CoV-2-infected HULEC-5a cells. **g** Representative images of peroxisomes and Golgi apparatus of mock- or SARS-CoV-2-infected HULEC-5a cells. All results are representative for three independent experiments.

For identification of infected cells, HPAEpiC cells were counterstained for viral Spike protein. In mock-infected HPAEpiC cells, endoplasmic reticulum exhibited a regular network structure and was distributed throughout the cytoplasm. However, in infected cells, the endoplasmic reticulum network was disrupted with some endoplasmic reticulum showing co-localization with viral particles (Fig. 2a). Similarly, Golgi apparatus and mitochondria also exhibited dramatic changes in the infected cells. Viral infection caused a significant decrease in the amount of Golgi apparatus (Fig. 2b) and over-fragmentation of mitochondria (Fig. 2c). Besides, peroxisomes were also examined, and the results showed SARS-CoV-2 infection caused a mild increase in size and a significant increase in density (number/cell) of peroxisomes (Fig. 2d, e). These findings indicated SARS-CoV-2 infection has a broad impact on organelle structures in HPAEpiC cells. The virus may hijack the host cell by remodeling its organelles for virus replication and spread.

In parallel, HULEC-5a cells were also carefully examined following viral infection. However, no obvious changes of these organelles were detected in the cells following viral infection (Fig. 2f, g), possibly due to low susceptibility of this cell type to SARS-CoV-2 infection.

**Fig. 3:**
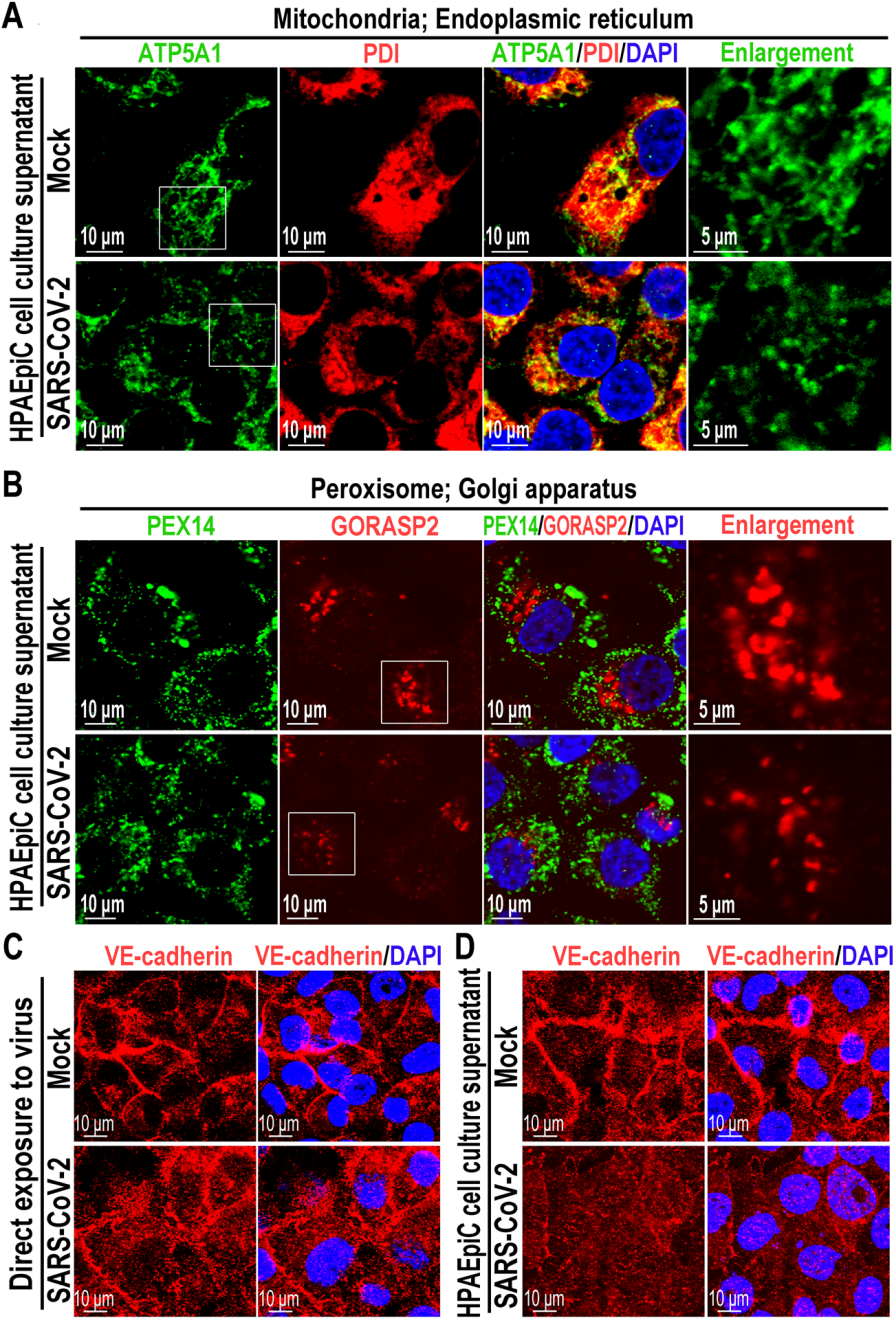
Organelles of HULEC-5a cells following treatment with culture supernatants from mock- or SARS-CoV-2-infected HPAEpiC cells. **a** Representative images of mitochondria and endoplasmic reticulum in HULEC-5a cells treated with mock- or SARS-CoV-2-infected HPAEpiC cell culture supernatants. The mitochondria indicated by white box were enlarged on the right. **b** Representative images of peroxisomes and Golgi apparatus in HULEC-5a cells treated with mock- or SARS-CoV-2-infected HPAEpiC cell culture supernatants. The Golgi apparatus indicated by white box was enlarged on the right. **c** Representative images of adherent junctions visualized by VE-cadherin in Mock- or SARS-CoV-2-infected HULEC-5a cells. **d** Representative images of adherent junctions visualized by VE-cadherin in HULEC-5a cells treated with mock- or SARS-CoV-2-infected HPAEpiC cell culture supernatants. All results represent three independent experiments.

As direct viral exposure had minimal effects on microvascular endothelial cells, we then hypothesized whether SARS-CoV-2 infection can induce alveolar epithelial cells to release some substances (such as cytokines), and further affects microvascular endothelial cells indirectly. To verify the hypothesis, culture supernatants were collected from mock- or SARS-CoV-2 infected HPAEpiC cells respectively and diluted with the same volume of fresh HULEC-5a cell medium, and then were utilized to treat HULEC-5a cells. 72 h later, HULEC-5a cells were examined by confocal microscope. The results showed, treatment of culture supernatant of SARS-CoV-2-infected HPAEpiC cells caused obvious mitochondrial fragmentation (Fig. 3a) and decrease of Golgi apparatus (Fig. 3b) in HULEC-5a cells. As the function of microvascular endothelial barrier depends heavily on the proper localization of adherent junction proteins, we then examined the adherent junctions of HULEC-5a cells by visualization of VE-cadherin. The results showed, direct exposure to SARS-CoV-2 had no obvious effect on VE-cadherin (Fig. 3c); but treatment of culture supernatant from infected HPAEpiC cells disrupted the adherent junction in HULEC-5a cells, as indicated by altered VE-cadherin organization (Fig. 3d). These findings suggested SARS-CoV-2 infection affected HULEC-5a cells in an indirect manner, possibly by substances secreted from infected HPAEpiC cells.

### SARS-CoV-2 infection in an alveolar epithelium/endothelium co-culture system

**Fig. 4:**
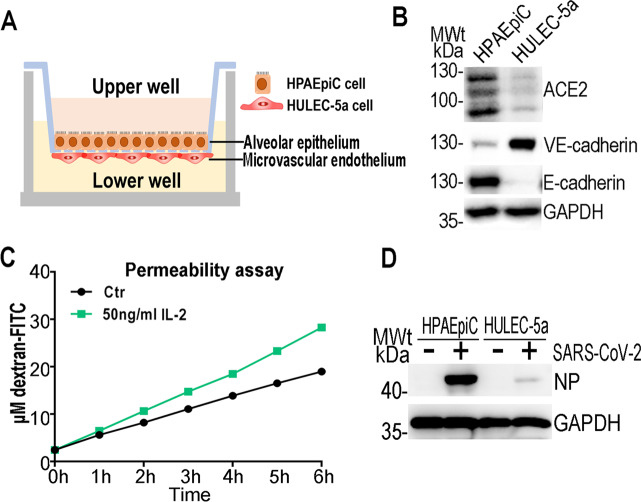
SARS-CoV-2 infection in an alveolar epithelium/endothelium co-culture system based on transwell. **a** A schematic diagram for an alveolar epithelium/endothelium co-culture system based on a transwell chamber. **b** Western blot was performed to analyze the levels of ACE2, E-cadherin (epithelial cell marker), and VE-cadherin (vascular endothelial cell marker) for HPAEpiC cells and HULEC-5a cells collected from the co-culture system, separately. **c** Blood-to-alveolus permeability assay of dextran-FITC (10 kDa) was measured on the co-culture system to evaluate the integrity of the alveolar–capillary barrier. Data were presented as mean ± SEM, *n* = 3. Data were analyzed by Student’s *t* test (***: *P* < 0.001). **d** Western blot was performed to analyze the viral Nucleoprotein (NP) level of HPAEpiC cells and HULEC-5a cells collected from the co-culture system separately following SARS-CoV-2 infection. **b**, **d** These results are representative blots from three independent experiments.

To investigate the responses of alveolar epithelium and pulmonary microvascular endothelium during SARS-CoV-2 infection in a near-physiological manner, we constructed an alveolar epithelium/endothelium co-culture system based on transwell (Fig. 4a). In this co-culture system, HPAEpiC cells and HULEC-5a cells were cultured on the upper and lower side of polycarbonate membrane to mimic the structure of alveolar–capillary barrier (Fig. 4a). To evaluate the condition of cells on this co-culture system, two types of cells were collected from each side of the membrane separately after three days co-culture, and then subjected to Western blot analysis. The result showed, over the course of three-day co-culture, two types of cells grew well and did not migrate to the opposite side of the membrane (HPAEpiC cells themselves express a very low-level VE-cadherin) (Fig. 4b). In addition, permeability assay of dextran-FITC (10 kDa) was performed to evaluate the integrity of alveolar–capillary barrier in this co-culture system. As shown in Fig. 4c, this co-culture system could maintain the integrity of barrier with a lower diffusion of dextran-FITC along the blood-to-alveolus direction, and this barrier integrity can be damaged by adding cytokine, such as IL-2.

To model SARS-CoV-2 infection in human alveolus, virus was inoculated in the upper well at an MOI of 10. After 72 h, cell samples were collected from transwell chambers and analyzed by Western blot to detect the viral Nucleocapsid protein (NP) expression level. The results showed, the NP protein level in HPAEpiC cells were much higher than that in HULEC-5a cells (Fig. 4d), which verified the previous finding that HPAEpiC cells were more permissive to viral infection than HULEC-5a cells (Fig. 1b–d). A histopathologic research revealed a similar finding that viral NP protein expression was prominently detected on alveolar epithelial cells and was minimally detectable on blood vessels within human lungs of COVID-19 deaths^3^.

### Define the proteomic response of host cells to SARS-CoV-2 infection

**Fig. 5:**
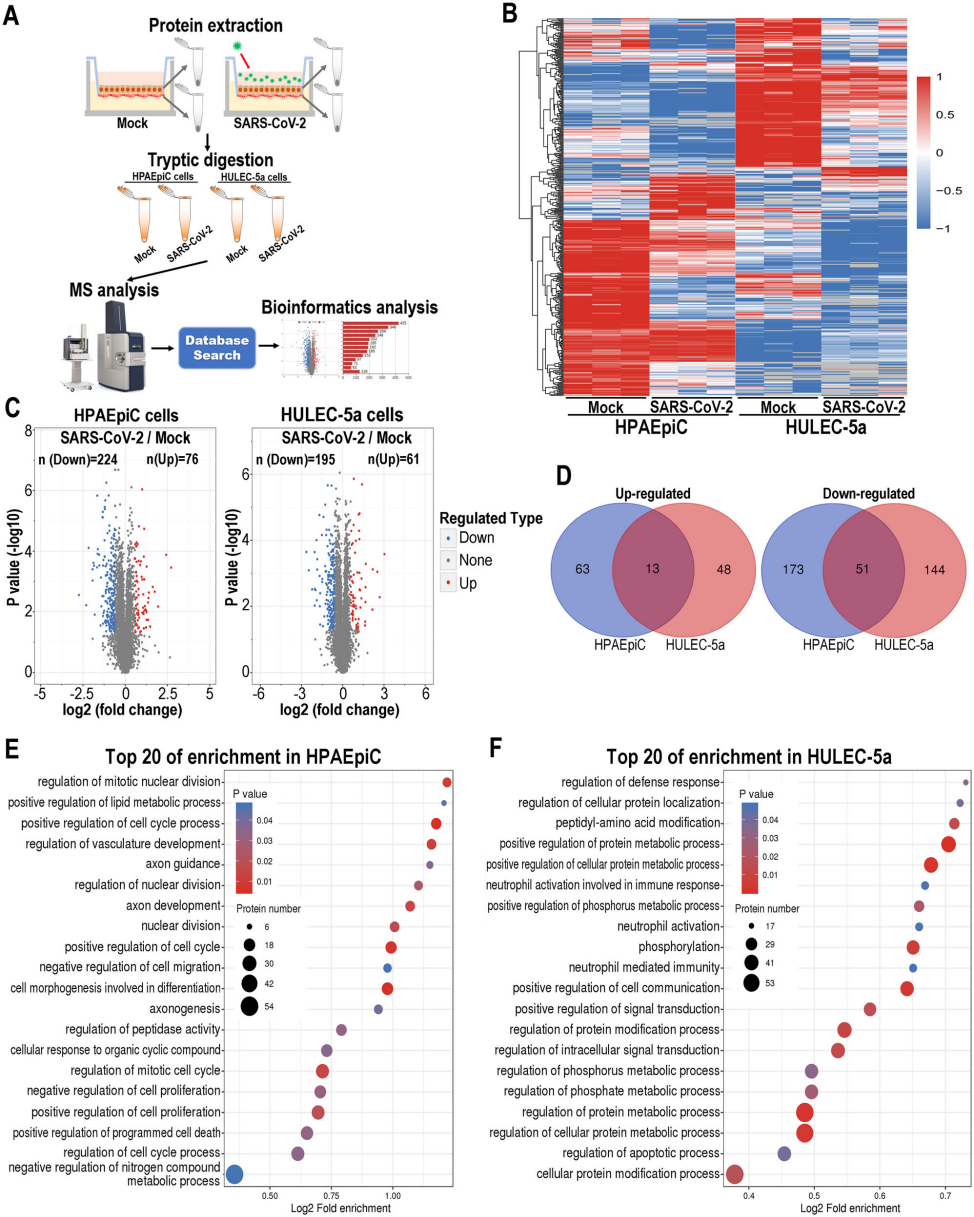
Proteomic analysis of host cells following SARS-CoV-2 infection based on alveolar co-culture system. **a** Schematic diagram of experimental flow for quantitative proteomic analysis based on the alveolar co-culture system. For each treatment, three biological replicates were prepared. **b** Heatmap showed proteomic changes of cells following SARS-CoV-2 infection. **c** Volcano plots showed the regulated proteins of cells following SARS-CoV-2 infection. Proteins differentially expressed with fold change over 1.5 and *p* < 0.05 were marked in color. *P*-values were calculated using a two-sided, unpaired Student’s *t* test with equal variance assumed (*n* = 3 independent biological samples). **d** Venn diagrams showed the differentially expressed proteins shared or unique between each comparison. **e** Gene Ontology enrichment analysis of DEPs in HPAEpiC cells following SARS-CoV-2 infection based on biological process. **f** Gene Ontology enrichment analysis of DEPs in HULEC-5a cells following SARS-CoV-2 infection based on biological process. **e**, **f** The vertical axis shows the top 20 enriched biological processes, and the horizontal axis represents rich factor. The color and size of the dots represent the range of the *P*-value and the number of DEGs mapped to the indicated GO terms, respectively. A two-tailed Fisher’s exact test was employed to test the enrichment of the differentially expressed protein against all identified proteins. The GO with a corrected *p*-value < 0.05 is considered significant.

To gain a comprehensive, unbiased overview of host responses of global proteins to viral infection, we performed a quantitative proteomic analysis based on this co-culture system. Briefly, 72 h after infection, HPAEpiC cells and HULEC-5a cells were collected separated from the co-culture system and then were analyzed by liquid chromatography–tandem mass spectrometry (LC–MS/MS) combined with advanced bioinformatics (Fig. 5a). Here, we adopted a quantitative, label-free proteomic approach based on trapped ion mobility spectrometry (TIMS) time-of-flight (TOF) pro, introduced by Bruker Inc. in 2017. This approach utilizes a quadruple parallel accumulation serial fragmentation acquisition method by synchronizing MS/MS precursor selection with TIMS separation, which can increase overall depth and resolution of the proteomic data with low sample amounts^24,25^.

Totally, 6546 proteins were identified, of which 6011 proteins were quantifiable. Heatmap showed SARS-CoV-2 infection-induced broad modulations of proteomes in both HPAEpiC cells and HULEC-5a cells (Fig. 5b). For identification of differentially expressed proteins (DEPs), the cutoffs for the fold change of abundance and *P*-value were set to 1.5 and 0.05, respectively. Proteins that did not reach these threshold parameters were not considered in further analysis. Among the DEPs, 300 proteins (76 up-regulated proteins and 224 down-regulated proteins) were significantly modulated in the infected HPAEpiC cells (Fig. 5c; Supplementary Table 1); while, 256 proteins (61 up-regulated proteins and 195 down-regulated proteins) were significantly modulated in the infected HULEC-5a cells (Fig. 5c; Supplementary Table 2). By combining the two datasets, we found just 64 DEPs, ~1% of total proteins quantified, were commonly regulated in both HPAEpiC cells and HULEC-5a cells following SARS-CoV-2 infection (Fig. 5d).

To investigate the host functions modified by SARS-CoV-2 infection, Gene Ontology enrichment analysis was performed to identify biological processes enriched among significantly regulated proteins. The results showed distinctive GO terms were enriched between HPAEpiC cells and HULEC-5a cells following viral infection (Fig. 5e, f). Among the top 20 of GO term enrichment, biological processes associated with cell cycle, cell proliferation, and regulation of programmed cell death were particularly enriched in HPAEpiC cells. While, in HULEC-5a cells, biological processes involved in protein metabolic process, neutrophil-mediated immunity, and signal transduction were significantly modulated.

Given a high viral load detected in alveolar epithelial cells, we then explored the effects of SARS-CoV-2 infection on immune or antiviral responses, by identifying significantly up-regulated proteins related to immune responses among DEPs of HPAEpiC cells (GO annotation keywords: immune response or antiviral response) (Supplementary Fig. 1). Notably, we found IL1α (a cytokine) and ISG15 (an interferon-induced protein) were up-regulated in SARS-CoV-2-infected HPAEpiC cells, which indicated some innate immune pathways were activated following viral infection. Moreover, we speculated the effects of viral infection on HULEC-5a cells were possibly mediated by cytokines secreted from infected-HPAEpiC cells.

### Proteomic analysis revealed mitochondrion was a primary target organelle for SARS-CoV-2 in host cells

**Fig. 6:**
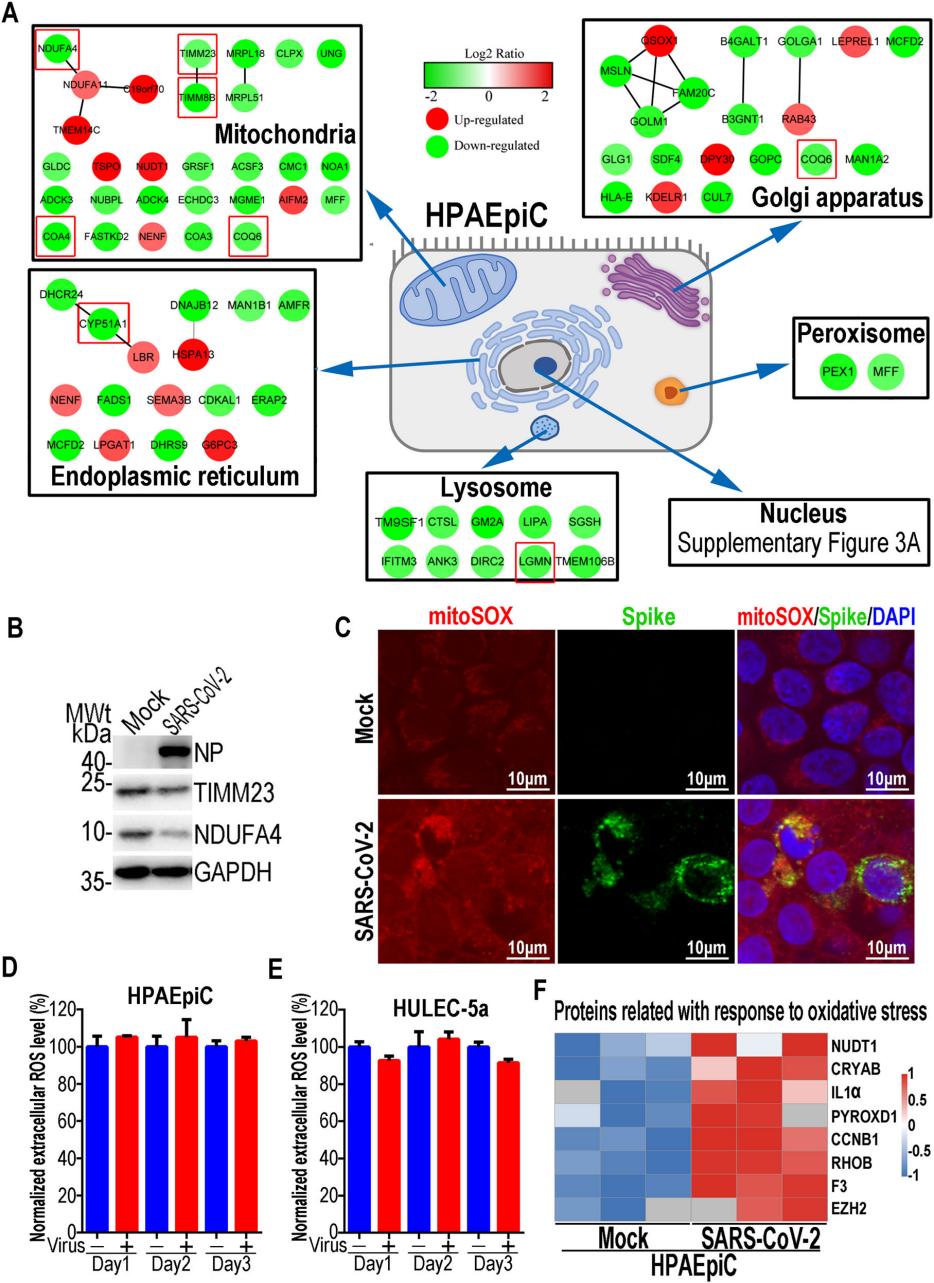
Mitochondrion was a primary target organelle by SARS-CoV-2 in host cells. **a** PPI (protein–protein interaction) networks of the DEPs within different sub-cellular compartments in HPAEpiC cells following SARS-CoV-2 infection. The red boxes indicated the DEPs shared in both HPAEpiC and Caco-2 cells following SARS-CoV-2 infection. Data of Caco-2 cells (24 h after infection) was from published literature by Bojkova et al.^26^. Proteins differentially expressed in Caco-2 (24 h post infeciton) with fold change over 1.3 and *p* < 0.05 were identified as DEPs (Supplementary Table 3). The color represents the log_2_ ratio. PPI network was constructed based on STRING database with the interaction score set to high confidence (0.700). **b** Western blot was performed to analyze the protein levels of TIMM23 and NDUFA4 in mock- and SARS-CoV-2-infected HPAEpiC cells. The result is a representative blot from three independent experiments. **c** Confocal fluorescent images of Mock- or SARS-CoV-2-infected HPAEpiC cells stained by mitoSox Red dye. Viral Spike protein indicated the infected cells. **d**, **e** Culture supernatants of HPAEpiC cells or HULEC-5a cells were harvested at indicated time points following SARS-CoV-2 infection to examine the extracellular ROS level using an ELISA kit for different groups. The results represent three independent experiments. Data were presented as mean ± SEM. The extracellular ROS level of mock-infected group at each time point was normalized to 1. **f** Heatmap showed the up-regulated proteins related to responses to oxidative stress in HPAEpiC cells following SARS-CoV-2 infection. Color indicates the abundance of a protein. All up-regulated proteins were searched based on GO annotation keywords (response to oxidative stress or response to reactive oxygen species). Fold change >1.5, *P*-value < 0.05 in (**d**).

As results above (Fig. 2a–e) have indicated SARS-CoV-2 can affect host cells by remodeling their organelles. We then performed sub-cellular distribution analysis of DEPs by cellular component annotation using UniProt database, to identify which organelles were significantly modulated by SARS-CoV-2 infection. The results showed, in both HPAEpiC cells and HULEC-5a cells, except for the nucleus, the largest proportion of DEPs were located in mitochondria, followed by endoplasmic reticulum and Golgi apparatus (Supplementary Fig. 2). Furthermore, protein–protein interaction (PPI) analysis of DEPs annotated to distinct sub-cellular compartments was performed to determine the potential relationships between DEPs. PPI network showed proteins within nucleus, mitochondria, endoplasmic reticulum, and Golgi apparatus underwent dramatic changes following viral infection in both cell types, and some DEPs were associated with at least one other protein (Fig. 6a; Supplementary Fig. 3).

We reasoned that, the regulated proteins which are essential for vital replication would be common in different cell types. As HULEC-5a cell itself is not susceptible to SARS-CoV-2 infection, we decided to choose a more susceptible cell line to identify commonly regulated proteins across different host cells. According to recent clinical and pathological studies, except for lung, intestine is another target organ for SARS-CoV-2. Caco-2 cell is a human colorectal adenocarcinoma cell line, and proteomic study on SARS-CoV-2-infected Caco-2 cells was published recently^26^. By combining the two data sets, we identified 3 up-regulated proteins and 10 down-regulated DEPs shared in both Caco-2 cells and HPAEpiC cells following SARS-CoV-2 (Supplementary Fig. 4A, B). Intriguingly, among them, 5 proteins were located on mitochondria (commonly regulated proteins were marked by red boxes in Fig. 6a). NDUFA4 is a subunit of complex IV of the electron transport chain, and COA4 is a putative assembly factor of complex IV^27–29^. TIMM23 is a mitochondrial inner membrane protein essential for import of preproteins into the matrix^30,31^. According to antibodies available, we performed Western blot to verify protein changes in HPAEpiC cells and the result showed NDUFA4 and TIMM23 were indeed down-regulated after viral infection (Fig. 6b).

Mitochondrion is the main producer of intracellular reactive oxygen species (ROS), and mitochondrial dysfunction causes excessive ROS production, which is detrimental for cells^32–34^. We then detected the mitochondrial ROS levels of mock- or SARS-CoV-2-infected HPAEpiC cells stained by mitoSOX Red fluorescent dye (a mitochondrial ROS indicator). As Fig. 6c showed, compared with mock-infected HPAEpiC cells, mitochondrial ROS level was significantly elevated in SARS-CoV-2-infected cells, especially the cells detected by viral Spike protein. It has been reported that ROS can diffuse from already damaged or activated cells to cells nearby, acting as an intercellular messenger^35,36^. Therefore, we tested the extracellular ROS level for culture supernatants of HPAEpiC cells and HULEC-5a cells following viral infection by an ELISA kit. The results showed viral infection caused a very mild increase of extracellular ROS level (*p* > 0.05) in HPAEpiC cells (Fig. 6d); while viral infection had no effect on extracellular ROS level in HULEC-5a cells at all (Fig. 6e). This result indicated ROS may not be the intercellular messenger that mediated the injury of HULEC-5a cells. At the meanwhile, proteomic analysis showed eight proteins annotated to responses to oxidative stress were significantly up-regulated in HPAEpiC cells following viral infection (Fig. 6f). Among the 8 modulated proteins, we identified IL1α which was annotated to response to reactive oxygen species (GO:0000302). According to published papers, IL1α can be up-regulated by excessive ROS^37,38^. Here, we proposed a hypothesis that the up-regulation of IL1α may be resulted from over-production of mitochondrial ROS, and IL1α may be secreted to extracellular space and further induced immune response in adjacent microvascular endothelial cells (HULEC-5a cells).

### Ultrastructural analysis of host cells following SARS-CoV-2 infection

To verify the proteomic analysis above, we performed ultrastructural examinations for HPAEpiC cells and HULEC-5a by transmission electron microscope (TEM), respectively. Same as previous experimental flow, virus was inoculated in the epithelial side, and 72 h later, HPAEpiC cells and HULEC-5a cells were collected separately for TEM examination.

**Fig. 7:**
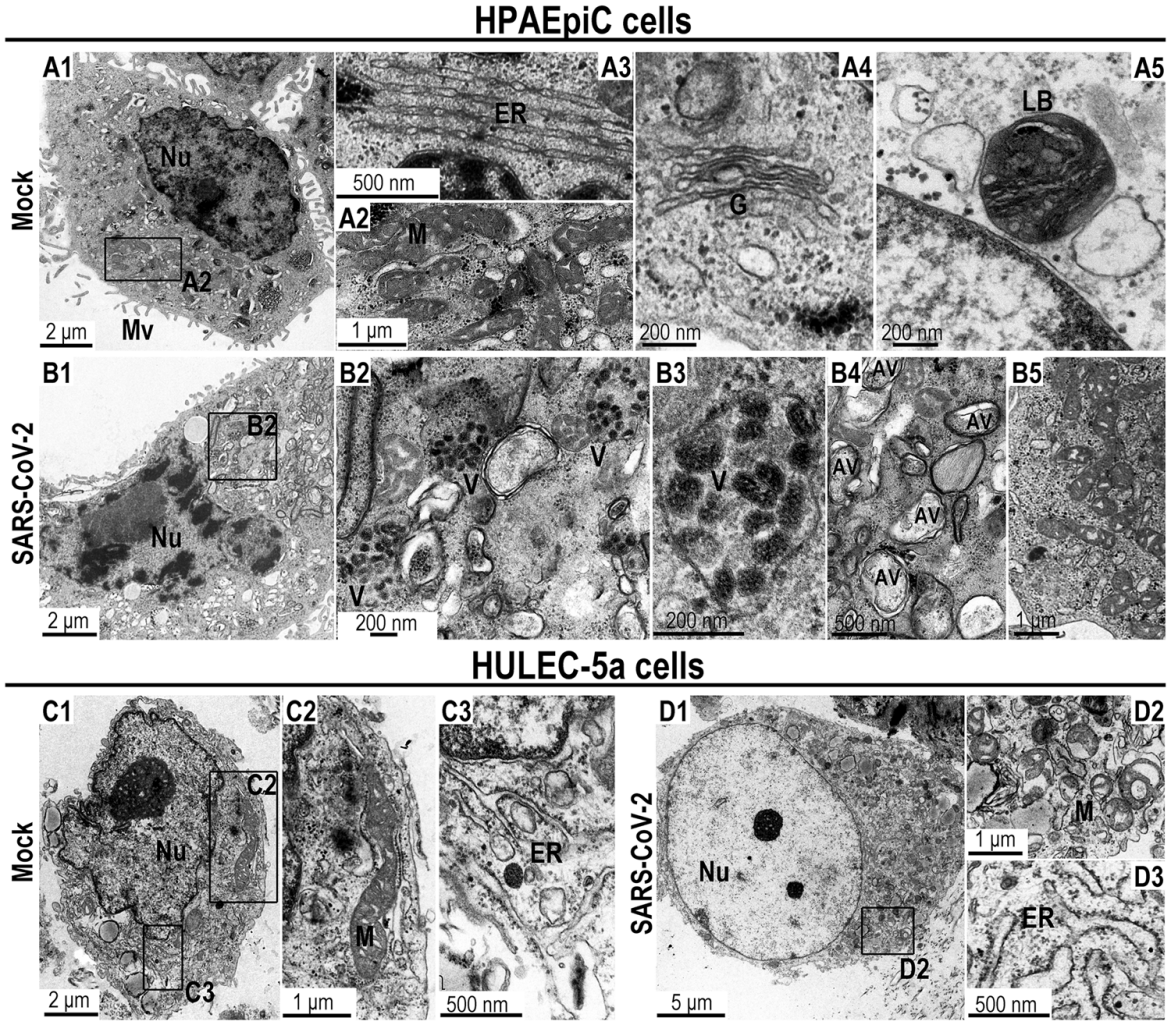
TEM analysis of mock- and SARS-CoV-2-infected cells from alveolar co-culture system. **a** TEM micrographs of HPAEpiC cells of mock-infected group. **a1** The overall image of the HPAEpiC cell. The area indicated by black box was enlarged in **a2**. **a2** The micrograph of mitochondria (M) of HPAEpiC cell. **a3** The micrograph of smooth endoplasmic reticulum (ER) of HPAEpiC cell. **a4** The micrograph of Golgi apparatus (G) of HPAEpiC cell. **a5** The micrograph of lamellar body (LB) within cell body. **b** TEM micrographs of HPAEpiC cells of SARS-CoV-2-infected group. **b1** The overall image of the infected cell. The area indicated by the black box is enlarged in **b2**. **b2** The micrograph of clusters of virus particles (V) within cell body. **b3** Enlarged image of virus indicated in **b2**. **b4** A large amount of autophagic vacuoles (AV) in the infected HPAEpiC cells. **b5** The micrograph of fragmented mitochondria (M) in the infected HPAEpiC cells. **c** TEM micrographs of HULEC-5a cells of mock-infected group. **c1** The overall image of the HULEC-5a cells. The areas indicated by black boxes were enlarged in **c2** and **c3**, respectively. **c2** The micrograph of mitochondria in the mock-infected HULEC-5a cells. **c3** The micrograph of rough endoplasmic reticulum (ER) in mock-infected HULEC-5a cells. **d** TEM micrographs of HULEC-5a cells of SARS-CoV-2-infected group. **d1** The overall image of the HULEC-5a cells. The areas indicated by black box were enlarged in **d2**. **d2** The micrograph of fragmented mitochondria (M) with swollen cristae in HULEC-5a cells of SARS-CoV-2-infected group. **d3** The micrograph of dilated rough endoplasmic reticulum (ER) in HULEC-5a cells of SARS-CoV-2-infected group. Nu: nucleus; Mv: microvilli; M: mitochondria; ER: endoplasmic reticulum; G: Golgi apparatus; LB: lamellar body; V: virus particle; AV: autophagic vacuole. Three independent experiments were performed (*n* = 3).

In mock-infected group, HPAEpiC cells exhibited a primary AT2-like structural feature^39,40^, such as a square or a circle cell shape, numerous microvilli on cell-free surface (Fig. 7a1) and lamellar bodies with high electron density (Fig. 7a5). Other types of organelles with regular morphology can also be observed, including mitochondria (Fig. 7a2), endoplasmic reticulum (Fig. 7a3) and Golgi apparatus (Fig 7a4). Contrastively, in SARS-CoV-2-infected group, HPAEpiC cells generally exhibited abnormal structural characteristics, with a large amount of vacuolar structures detected in the infected cells (Fig. 7b1). Virus particles (~100 nm diameter) were distributed in clusters within cytoplasm, and many virus particles were encapsulated in cytoplasmic vesicles (Fig. 7b2, b3), which is similar to reports in lung samples of COVID-19 deaths^41^. Moreover, co-existing with virus particles, lots of autophagic vacuoles (Fig. 7b4) and fragmented mitochondria (Fig. 7b5) were detected.

Different from HPAEpiC cells, no virus particles were detected in HULEC-5a cells, possibly due to low viral load. However, many organelles with abnormal morphology were also detected in HULEC-5a cells following alveolar epithelial infection, such as fragmented mitochondria with swollen cristae and rough endoplasmic reticulum with dilated lumen (Fig. 7c, d).

Collectively, the EM data showed SARS-CoV-2 infection caused a dramatic re-organization of organelles in HPAEpiC cells, accompanied by a robust viral replication. While, in HULEC-5a cells, despite no virus particles detected, many organelles still exhibited abnormal morphology, which indicated again that SARS-CoV-2 infection affected pulmonary microvascular endothelial cells in an indirect manner, possibly mediated by infected alveolar epithelial cells.

### Daurisoline, an autophagy inhibitor, could inhibit SARS-CoV-2 replication in host cells

**Fig. 8:**
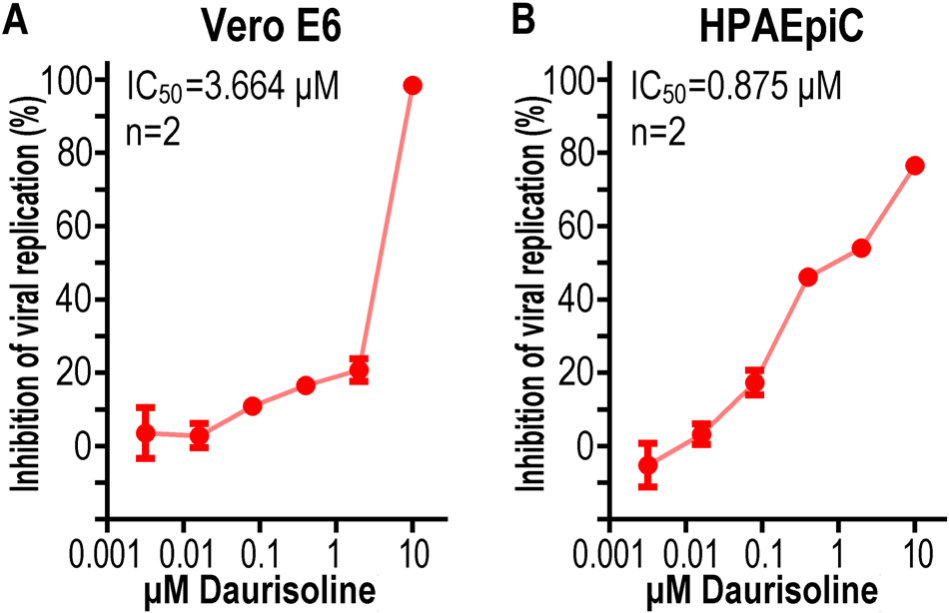
Daurisoline could inhibit SARS-CoV-2 replication in Vero E6 cells and HPAEpiC cells. **a** Antiviral assay showed inhibition of viral replication in dependency of Daurisoline concentration in Vero E6 cells (*n* = 2). **b** Antiviral assay showed inhibition of viral replication in dependency of Daurisoline concentration in HPAEpiC cells (*n* = 2).

As detected by electron microscope, there are more autophagosomes in the infected HPAEpiC cells, we then hypothesized the autophagy pathway may be involved in SARS-CoV-2 infection. Quantitative proteomic analysis showed^19^ autophagy-related proteins were significantly modulated in HPAEpiC cells following SARS-CoV-2 infection (Supplementary Fig. 5). Therefore, we investigated whether inhibition of autophagy pathway could interfere viral replication in host cells. Firstly, we tested six autophagy blockers (Supplementary Table 4), and found one of them, Daurisoline, could significantly inhibit virus replication in Vero E6 cells (Fig. 8a). Subsequently, we validated the antiviral effect of Daurisoline in lung-related cells, and the result showed Daurisoline could inhibit virus replication effectively in HPAEpiC cells as well (Fig. 8b). Daurisoline (a bis-benzylisoquinoline alkaloid isolated from the rhizomes of Menispermum dauricum) is a potent autophagy blocker with antiarrhythmic effects, and has been studied for treatment of arrhythmia^42,43^.

## Discussion

In the present study, we focused on defining the effects of SARS-CoV-2 infection on alveolar epithelium and pulmonary microvascular endothelium, and sought to elucidate the mechanism of alveolar–capillary injury during COVID-19. In general, our data showed SARS-CoV-2 infection-induced distinctive responses in epithelium and endothelium, including susceptibility to viral infection, host organelles remodeling, proteome modulations and ultrastructural changes.

SARS-CoV-2 infection caused a dramatic remodeling of organelles in epithelial cells alone, including mitochondria, Golgi apparatus, endoplasmic reticulum and peroxisomes. Notably, we found some endoplasmic reticulum showed co-localization with viral particles in infected epithelial cells. Based on a research of SARS-CoV, nucleocapsids of SARS-CoV are firstly assembled within lumen of rough endoplasmic reticulum during viral replication^44^. Given SARS-CoV-2 is closely related to SARS-CoV on evolution, SARS-CoV-2 may have a similar life cycle to SARS-CoV. Besides, we also detected an increase of peroxisomes in the infected epithelial cells. Peroxisome is a metabolic organelle with vital functions in deactivation of toxic substances and lipid metabolism^45^. A study reported enveloped viruses, such as human cytomegalovirus and herpes simplex virus type 1, can induce the biogenesis of peroxisomes for phospholipid plasmalogen synthesis, thereby enhancing virus production^45^. As SARS-CoV-2 has an envelope, and the increased peroxisomes may be related to virus replication as well. While, direct viral exposure had no obvious effect on pulmonary microvascular endothelial cells. But, treatment with culture supernatant from infected alveolar epithelial cells seriously injured the endothelial cells, including mitochondrial over-fragmentation, decreased Golgi apparatus and disrupted adherent junctions. This finding suggested SARS-CoV-2 injured pulmonary microvascular endothelium in an indirect manner, possibly by substances released from alveolar epithelium.

Virus itself is not an independent living entity, and it relies heavily on host machineries to achieve its replication and spread^13,14^. Therefore, it’s key to understand how virus usurps host cell at the molecular level. Here, we adopted a quantitative proteomic approach to explore the global protein changes of host cells following SARS-CoV-2 infection. Proteomic analysis showed viral infection elicited a broad protein changes in both cell types, but the modulated biological processes were distinctive between them. Among the significantly up-regulated proteins in alveolar epithelial cells, we identified some proteins related to immune responses (e.g. IL1α and an interferon-induced protein ISG15). Considering the lower viral load in microvascular endothelial cells, we speculated SARS-CoV-2 affected pulmonary microvascular endothelium possibly by cytokines (e.g. IL1α and IFNs) released from the infected alveolar epithelium.

As our study has indicated SARS-CoV-2 infection can affect host cells by remodeling their organelles, sub-cellular analysis of DEPs was then performed to analyze how virus hijacks and re-organizes host organelles at the molecular level. The results showed SARS-CoV-2 infection induced a broad protein changes across many organelles, especially mitochondria, endoplasmic reticulum and Golgi apparatus in both cell types. Given the vital roles of these organelles in energy metabolism, protein and lipid synthesis, disturbance of these organelles may significantly affect the host cells. We reasoned the regulated proteins which were important for viral replication would be common to different cell types. By combining our proteomic data with published data^26^, we identified 13 overlapping DEPs which were shared in both HPAEpiC cells and Caco-2 cells following SARS-CoV-2 infection. Intriguingly, among them, five proteins were located on mitochondria. This finding indicated mitochondrion may be a commonly hijacked organelle by SARS-CoV-2 across different infected organs. Western blot showed, two of the five commonly modulated mitochondrial proteins, NDUFA4 and TIMM23 were down-regulated in HPAEpiC cells after SARS-CoV-2 infection. At the meanwhile, fluorescent assay indicated mitochondrial ROS level was elevated in the infected cells. According to published papers, elevated ROS can induce expression of IL1α^37,38^. Here, we proposed a hypothesis that SARS-CoV-2 infection disrupted mitochondria and caused ROS over-production, and excessive ROS could induce IL1α expression and secretion to extracellular space, which further affected adjacent microvascular endothelial cells.

Consistent with proteomic analysis, EM examinations showed SARS-CoV-2 infection caused obvious ultrastructural changes in both cell types, although virus particles were hardly detected in microvascular endothelial cells. This result indicated again that SARS-CoV-2 infection can injure pulmonary microvascular endothelium in the absence of viral replication. Notably, we detected more autophagic vacuoles in the infected epithelial cells. Proteomic analysis also showed many autophagy-related proteins were modulated in epithelial cells following viral infection. We tested several autophagy inhibitors, and the result showed Daurisoline could effectively inhibit viral replication at cellular level. Of course, this is just a preliminary result, which remains to be further tested by animal trials.

Our studies explored the effects of SARS-CoV-2 infection on alveolar epithelial cells and pulmonary microvascular endothelial cells, and revealed a cross-talk between epithelium and endothelium during viral infection. This cross-talk mediated microvascular endothelial injury. This finding may help us understand the injury mechanism of alveolar–capillary barrier, and may also provide some clues for target drug development.

## Materials and methods

### Cell culture

Vero E6 cells were obtained from American Type Culture Collection (ATCC, no. 1586) and maintained in MEM medium (Gibco) supplemented with 10% fetal bovine serum (FBS; Gibco). Immortalized human alveolar epithelial cells (HPAEpiCs) were generated from Type II pneumocytes of human lung tissue (purchased form Sciencell Shanghai Corporation) and are maintained in RPMI 1640 medium (Gibco) supplemented with 10% FBS (Gibco) and 1% P/S. HULEC-5a cells were purchased from Procell Corporation (introduced from ATCC Company), and are maintained in HULEC-5a growth medium (Procell, CM-0565). All cells were cultured at 37 °C in a humidified atmosphere of 5% CO_2_. All cell lines used in this study authenticated by STR profiling and were tested negative for mycoplasma contamination.

### Virus preparation and viral titer determination

A clinical isolate SARS-COV-2 strain 107, which was obtained from Guangdong Provincial Center for Disease Control and Prevention, Guangdong Province of China, was propagated in Vero E6 cells, and viral titer was determined by a TCID50 assay on Vero E6 cells. All the viral experiments were performed in a biosafety level-3 (BSL-3) laboratory.

### SARS-CoV-2 infections

Cells were seeded in 35 mm glass-bottom dish or 48-well plates, and 24 h later, cells were infected with SARS-CoV-2 at an MOI of 10. One hour after infection, cells were washed three times with PBS and cultured in fresh medium for 3 days. On day 3 post-infection, culture supernatant was collected for RNA extraction, or fixed with 4% PFA for fluorescent imaging.

For SARS-CoV-2 infection in the transwell, 1 mL of RPMI 1640 medium containing the indicated multiplicity of virus (MOI = 10) were added in the upper well. One hour after infection, cells in the upper well were washed three times with PBS and kept in fresh medium. On day 3 post-infection, cells were lysed for sample preparation of Western blot or quantitative mass spectrometry.

### Antibodies, reagents, and lab consumables

Anti-ACE2 antibody (21115-1-AP), anti-TMPRSS2 antibody (14437-1-AP), anti-E-cadherin antibody (60335-1-Ig), anti-VE-cadherin antibody (66804-1-Ig), anti-GORASP2 antibody (66627-1-Ig), anti-PDI antibody (66422-1-Ig), anti-ATP5A1 antibody (66037-1-Ig), anti-ATP5A1 antibody (14676-1-AP), anti-PEX14 antibody (10594-1-AP) and anti-TIMM23 antibody (11123-1-AP) were purchased from Proteintech Group. Anti-NDUFA4 antibody (YT3008) was purchased from Immunoway company. Anti-GAPDH (CW0100) antibody was purchased from CWBIO company. Anti-Spike antibody (40150-R007) and anti-Nucleoprotein antibody (40143-R019) were purchased from Sino Biological company.

The list of drugs and suppliers was shown in Supplementary Table 4. Lab consumables were bought from Guangzhou Jet Bio-Filtration Co., Ltd.

### Western blot analysis

Protein samples were separated on 10% SDS-PAGE and then transferred onto 0.2 μm nitrocellulose membranes (GE Amersham). After being blocked with 5% BSA in TBST buffer containing 0.05% Tween-20, the membranes were probed with the primary antibodies at 4 °C overnight. The membranes were then probed with corresponding horseradish peroxidase (HRP)-conjugated secondary antibodies at room temperature for 1 h at room temperature. Protein bands were detected by Prime Western Blotting Detection Reagent (GE life).

### Determination of virus titers using qRT-PCR

One hundred microliter of cell culture supernatant was harvested for viral RNA extraction using the HP Viral RNA Kit (Roche, Cat no. 11858882001) according to the manufacturer’s instructions. qRT-PCR was performed on Real-Time PCR System (Applied Biosystem, ViiA™ 7) with One Step RT-PCR RNA direct realtime PCR mastermix (TOYOBO, QRT-101A). The primers for realtime PCR were as follows: N-F: 5′-GGGGAACTTCTCCTGCTAGAAT-3′; N-R: 5′-CAGACATTTTGCTCTCAAGCTG-3′; N-probe: 5′- TTGCTGCTGCTTGACAGATT-3′. PCR amplification was performed as follows: 50 °C for 10 min, and 95 °C 1 min followed by 45 cycles consisting of 95 °C for 15 s, 60 °C for 45 s.

### Immunofluorescent staining

Cells were fixed with 4% paraformaldehyde (PFA) at 4 °C overnight. Then, cells were washed with PBS, and then permeabilized and blocked with PBST buffer (0.2% Triton X-100 in PBS buffer) containing 5% normal goat serum for 30 min at room temperature. Antibodies were diluted with PBST buffer. Cells were immunostained with primary antibodies at 4 °C overnight and with secondary antibodies at room temperature for 1 h. After staining with antibodies, cells were counterstained with DAPI before mounting. Images were acquired using a Carl Zeiss LSM880 confocal fluorescent microscope system.

### Co-culture of cells on the transwell chamber

Cell co-culture was performed using transwell chambers (6 well plate) containing 0.4 μm pore filters (Corning Incorporated) according to a published protocol with modifications^46^. Before cell culture, transwell chambers were coated with type I collagen (1:50 dilution) for 48 h, with both sides of the polycarbonate membrane immersed in collagen solution. Wash the chambers three time with PBS before use. Pick the transwell insert out of the 6 well plate and put it upside down on a petri dish. HULEC-5a cells were inoculated on the opposite side of polycarbonate membrane at a density of 1 × 10^6^, and then were cultured in incubator for 2 h. Put the transwell insert back in the 6 well plate, and immersed the HULEC-5a cells in medium. 5 × 10^6^ HPAEpiC cells were inoculated on the upper well and were cultured for another 24 h before viral infection.

### Permeability assay of alveolar–capillary barrier

HULEC-5a cells and HPAEpiC cells were plated on transwells 24 h before the assay, and then expose the HULEC-5a cells to 50 ng/mL IL-2 for 4 days. Wash HPAEpiC cells and HULEC-5a cells with RPMI1640 medium for three times. After that, 100 μM dextran-FITC were added in the lower well (vascular channel). The concentration of dextran-FITC of upper well (alveolar channer) were recorded by microplate reader at various time points (0 h, 1 h, 2 h, 3 h, 4 h, 5 h, 6 h) to determine the permeability of alveolar–capillary barrier.

### Antiviral assay

For antiviral assay on Vero E6 cells, the antiviral effect of the tested drug on Vero E6 was determined by CCK8 assay (Beyotime, China). Briefly, Vero E6 cells were plated on 96-well plate 24 h before drug testing. Vero E6 cells were infected with SARS-CoV-2 at an MOI of 0.1. Virus was added together with drugs and incubated in MEM supplemented with 2% FBS with different drug dilutions. 1 h later, the virus–drug mixture was removed and cells were further cultured with fresh drug-containing medium. After 72 h, cell viability was determined by CCK8 assay, and the concentration required for an inhibitory of 50% (IC50) was calculated by the OriginPro2016 software.

For antiviral assay on HPAEpiC cells, the viral replication was determined by qPCR. Briefly, HPAEpiC cells were plated on 48-well plate 24 h before drug testing. HPAEpiC cells were infected with SARS-CoV-2 at a MOI of 10. Virus was added together with drugs and incubated in RPMI 1640 medium supplemented with 10% FBS with different drug dilutions. 1 h later, the virus–drug mixture was removed and cells were further cultured with fresh drug-containing medium. After 72 h, the cell supernatants were collected for RNA extraction and viral copy determination by qPCR.

### Mitochondrial ROS detection assay

HPAEpiC cells were seeded on 35 mm glass-bottom dish (2 × 10^6^ cells per dish) in RPMI 1640 medium containing 10% FBS. After seeding for 24 h, cells were infected with SARS-CoV-2 at a MOI of 10. One hour after infection, cells were washed three times with PBS and cultured in fresh medium for 3 days. On day 3 post-infection, cells were stained with mitoSOX Red for 20 min at 37 °C. After washed with PBS, cells were fixed with 4% paraformaldehyde (PFA) for 20 min at room temperature. Before observation by confocal microscope, cells were stained by DAPI and anti-Spike antibody.

### Transmission electron microscopy

HPAEpiC cells and HULEC-5a cells were collected separately from the transwell, and then and fixed in PBS buffer containing 4% PFA (Electron Microscopy Sciences) and 2.5% glutaraldehyde (Electron Microscopy Sciences) at 4 °C overnight. After washed with PBS three times and fixed in 1% OsO4 buffer for 2 h, the samples were dehydrated with graded ethanol solutions, and then embedded in Epon812 resin (SPI). Ultrathin sections (70 nm) were stained with 2% uranyl acetate for 30 min and then lead citrate for 10 min. Images were acquired by JEM-1400PLUS electron microscope.

### Protein extraction and trypsin digestion for quantitative mass spectrometry

Cell samples were collected from transwells, lysed in lysis buffer, and then inactivated by autoclave sterilization in BSL-3 lab. Immediately, protein samples were mailed in dry ice to Jingjie PTM BioLab (Hangzhou) Co. Ltd for mass spectrometry analysis. There, the samples were re-inactivated and then sonicated three times on ice using a high-intensity ultrasonic processor (Scientz) in lysis buffer. The remaining debris was removed by centrifugation at 12,000 *g* at 4 °C for 10 min. Finally, the supernatant was collected and the protein concentration was determined with a BCA kit according to the manufacturer’s instructions.

For tryptic digestion, the protein solution was reduced with 5 mM dithiothreitol for 30 min at 56 °C and alkylated with 11 mM iodoacetamide for 15 min at room temperature in darkness. The protein sample was then diluted by adding 100 mM TEAB to urea (less than 2 M). Finally, trypsin was added at 1:50 trypsin-to-protein mass ratio for the first digestion overnight and 1:100 trypsin-to-protein mass ratio for a second 4 h-digestion.

### Analysis by liquid chromatography-mass spectrometry

The tryptic peptides were dissolved in solvent A (0.1% formic acid, 2% acetonitrile/ in water), directly loaded onto a home-made reversed-phase analytical column (25-cm length, 75/100 μm i.d.). Peptides were separated with a gradient from 6% to 24% solvent B (0.1% formic acid in acetonitrile) over 70 min, 24% to 35% in 14 min and climbing to 80% in 3 min then holding at 80% for the last 3 min, all at a constant flow rate of 450 nL/min on a nanoElute UHPLC system (Bruker Daltonics).

The peptides were subjected to Capillary source followed by the timsTOF Pro (Bruker Daltonics) mass spectrometry. The electrospray voltage applied was 1.75 kV. Precursors and fragments were analyzed at the TOF detector, with a MS/MS scan range from 100 to 1700 *m/z*. The timsTOF Pro was operated in parallel accumulation serial fragmentation (PASEF) mode. Precursors with charge states 0 to 5 were selected for fragmentation, and 10 PASEF-MS/MS scans were acquired per cycle. The dynamic exclusion was set to 30 s.

### Database search

The resulting MS/MS data were processed using MaxQuant search engine (v.1.6.6.0). Tandem mass spectra were searched against the KA158LQ database (20395 entries) concatenated with reverse decoy database and common contamination database. Trypsin/P was specified as cleavage enzyme allowing up to two missing cleavages. The mass tolerance for precursor ions was set as 20 ppm in First search and 20 ppm in Main search, and the mass tolerance for fragment ions was set as 20 ppm. Carbamidomethyl on Cys was specified as a fixed modification, and acetylation on protein N-terminal and oxidation on Met were specified as variable modifications. FDR was adjusted to <1%.

### Proteome annotation

Gene Ontology (GO) annotation was performed based on the UniProt-GOA database (http://www.ebi.ac.uk/GOA/). Firstly, convert identified protein ID to UniProt ID and then mapping to GO IDs by protein ID. If some identified proteins were not annotated by the UniProt-GOA database, the InterProScan software v5.36 would be used to annotated protein’s GO functional based on the protein sequence alignment method. Protein domains were annotated by InterProScan based on the InterPro database (v75.0) with default parameters. Kyoto Encyclopedia of Genes and Genomes (KEGG) pathway database (https://www.genome.jp/kegg/pathway.html) was used to annotate the protein pathway. Firstly, the KEGG online service tool KAAS (https://www.genome.jp/kaas-bin/kaas_main) was used to annotate protein’s KEGG database accession. Then the annotation results were mapped using KEGG online service tool KEGG mapper (https://www.genome.jp/kegg/mapper.html). EuKaryotic Orthologous Groups were annotated with the KOG database proteins using BLASTp v2.2.31 with a maximal e-value of 1e-5. Sub-cellular localization predication was performed based on the UniProtKB database (https://www.uniprot.org/uniprot/).

### Identification of differentially expressed proteins (DEPs)

The quantitative values of each sample in three replicates were obtained by LFQ intensity. The first step is to calculate the differential expression of the protein between the two samples. Calculate the average value of the quantitative values of each sample in multiple replicates, and then calculate the ratio of the average values between the two samples. The ratio is used as the final quantitation. The second step is to calculate the significant *p*-value of differential expression between two samples. The relative quantitative values of each sample were taken as log2 transform (so that the data conforms to the normal distribution), and *p*-value was calculated by the two-sample two-tailed *T*-test method.

### Functional enrichment

For each of the GO and KEGG pathway, a double-tailed Fisher’s exact test was performed using the R package “stats” to detect the enrichment of differentially expressed proteins against all identified proteins. The GO categories and KEGG pathways with a revised *p*-value < 0.05 were considered significant.

### Enrichment-based clustering

Further hierarchical clustering was based on different protein functional classifications, including GO, Domain, and KEGG. All the categories were collated after enrichment along with their p-values, then those categories which were at least enriched in one of the clusters with p-value < 0.05 were filtered. This filtered p-value matrix was transformed by the function *x* = −log10 (*p*-value). Finally, those *x* values were *z*-transformed for each functional category. The *z* scores were then clustered by one-way hierarchical clustering (Euclidean distance, average linkage clustering) in Genesis. Cluster membership was visualized by a heatmap using the “heatmap.2” function from the “gplots” R-package.

### Protein–protein Interaction Network

All differentially expressed proteins belonging to different sub-cellular compartments were searched against the STRING database version 11.0 (https://string-db.org/) to retrieve their interactions. Only interactions between the proteins within the searched dataset were selected. STRING database defines a metric called “confidence score” to represent the interaction confidence. Here, we selected all interactions with a confidence score >0.7 (high confidence). The Interaction network from the STRING database was visualized by using the Cytoscape 3.8.0 software. Individual DEPs without interaction were added in the Cytoscape 3.8.0 software.

### Statistical analyses

Data were collected and organized by Excel (Microsoft) software. The GraphPad Prism 6 software was used for data statistical analysis. All experiments were performed at least three times. During the experiment and assessing the outcome, the investigators were blinded to the group allocation. Differences between two groups were analyzed using a Student’s *t* test. Multiple group comparisons were performed using a one-way analysis of variance (ANOVA) followed by Bonferroni post hoc test. The bar graphs with error bars represent mean ± standard error of the mean (SEM). Significance is indicated by asterisks: *, *P* < 0.05; **, *P* < 0.01; ***, *P* < 0.001.

## Supplementary information

Supplementary Figure Legends

Supplementary Figure 1

Supplementary Figure 2

Supplementary Figure 3

Supplementary Figure 4

Supplementary Figure 5

Supplementary Figure 6

Supplementary Table 1

Supplementary Table 2

Supplementary Table 3

Supplementary Table 4

## Data Availability

All relevant data are available in the manuscript or supplementary information. The mass spectrometry proteomics data have been deposited to the ProteomeXchange Consortium via the PRIDE partner repository with the dataset identifier PXD020470.
