# Supplementary material for: A cross-talk between epithelium and endothelium mediates human alveolar–capillary injury during SARS-CoV-2 infection
Source: Cell Death Dis. 2020 Dec 8;11(12):1042. doi: 10.1038/s41419-020-03252-9 (PMC7721862; doi:10.1038/s41419-020-03252-9)
Supplement: Supplementary file 1 — Supplementary Figure Legends [file 41419_2020_3252_MOESM1_ESM.docx]

[**Supplementary Figure Legends**](https://static-content.springer.com/esm/art%3A10.1038%2Fs41419-020-03191-5/MediaObjects/41419_2020_3191_MOESM1_ESM.docx)

Peng Wang^1,#^, Ronghua Luo^5,6,#^, Min Zhang^1, 4,#^, Yaqing Wang^1,4^, Tianzhang Song^5,6^, Zhongyu Li^1^, Lin Jin^7^, Hongyi Zheng^5,6^, Wenwen Chen^1,4^, Mengqian Zhao^1,4^, Tingting Tao^1,4^, Yongtang Zheng^4,5,6,^*, Jianhua Qin^1,2,3,4,*^

^1^Division of Biotechnology, CAS Key Laboratory of SSAC, Dalian Institute of Chemical Physics, Chinese Academy of Sciences, Dalian, China.

^2^Institute for Stem Cell and Regeneration, Chinese Academy of Sciences, Beijing, China

^3^CAS Center for Excellence in Brain Science and Intelligence Technology, Chinese Academy of Sciences, Shanghai, China

^4^University of Chinese Academy of Sciences, Beijing, China

^5^Key Laboratory of Animal Models and Human Disease Mechanisms of Chinese Academy of Sciences, KIZ-CUHK Joint Laboratory of Bioresources and Molecular Research in Common Diseases, Kunming Institute of Zoology, Chinese Academy of Sciences, Kunming, Yunnan 650223, China

^6^Kunming National High-level Bio-safety Research Center for Non-human Primates, Center for Biosafety Mega-Science, Kunming Institute of Zoology, Chinese Academy of Sciences, Kunming, 650107, China

^7^Key Laboratory of Animal Models and Human Disease Mechanisms of Chinese Academy of Sciences, Kunming Institute of Zoology, Chinese Academy of Sciences, Kunming 650223, Yunnan, China

#These authors contribute equally to this work.

***Correspondence:** Jianhua Qin, Division of Biotechnology, Dalian Institute of Chemical Physics, Chinese Academy of Sciences, 457 Zhongshan Road, Dalian 116023, China. E-mail: jhqin@dicp.ac.cn. Tel: 86-0411-84379650.

Yongtang Zheng, Kunming Institute of Zoology, Chinese Academy of Sciences, 32 Jiaochang Donglu Kunming, Yunnan, E-mail: zhengyt@mail.kiz.ac.cn. Tel: +86 0871 65295684.

**Supplementary Figure 1. Heatmap showed the up-regulated proteins related with immune responses or antiviral response in HPAEpiC cells following SARS-CoV-2 infection.** Color indicates the abundance of protein. Fold change>1.5, P value<0.05.

**Supplementary Figure 2. Sub-cellular distributions of the differentially expressed proteins in HPAEpiC cells and HULEC-5a following SARS-CoV-2 infection.** The number in bracket indicates the number of differentially expressed proteins in each subcellular compartment.

**Supplementary Figure 3. PPI networks of the differentially expressed proteins within different sub-cellular compartments in cells following SARS-CoV-2 infection. (A)** PPI network of the DEPs within nucleus in HPAEpiC cells following SARS-CoV-2 infection. **(B)** PPI networks of the DEPs within different sub-cellular compartments in HULEC-5a cells following SARS-CoV-2 infection. The color represents the log_2_ ratio. Individual DEPs without interacts were shown below. PPI network was constructed based on STRING database with the interaction score set to high confidence (0.700).

**Supplementary Figure 4. Comparison of DEPs between Caco-2 cells and HPAEpiC cells following SARS-CoV-2 infection. (A)** Venn diagrams showed the differentially expressed proteins shared or unique between Caco-2 cells and HPAEpiC cells following SARS-CoV-2 infection. Data of Caco-2 cells (24h) is from published literature by Bojkova, et al. ^1^. **(B)** DEPs list shared between Caco-2 cells and HPAEpiC cells following SARS-CoV-2 infection.

**Supplementary Figure 5. Heatmap showed the differentially expressed proteins related with autophagy in HPAEpiC cells following SARS-CoV-2 infection.** Color indicates the abundance of protein. Fold change>1.5, P value<0.05.

**Supplementary Figure 6. A proposed model for SARS-CoV-2-induced injuries of alveolar endothelial cells and epithelial cells.** When exposed to SARS-CoV-2, virus particles invade alveolar epithelial cells (especially AT2 cells), and target cellular machineries and usurp their functions for viral massive replication. During the process, infected epithelial cells undergo global proteome modulations and structural remodeling across many sub-cellular compartments, including nuclear, mitochondria, ER ([endoplasmic reticulum](javascript:;)), Golgi apparatus, peroxisomes and so on. Among the affected organelles, mitochondria seem to be a primary target for virus. SARS-CoV-2 infection dys-regulates some critical proteins within mitochondria (e.g. NDUFA4 and TIMM23), and causes mitochondrial dysfunction and ROS over-production. Elevated ROS induces oxidative stress and may further up-regulate IL-1α. At the meanwhile, virus infection may activate INFs-mediated antiviral responses by up-regulating IFNs in alveolar epithelial cells. Proinflammatory cytokines, such as IL-1α and IFNs, can be released to extracellular space, and further induces damage (immune responses) of adjacent microvascular endothelial cells.

**Reference:**

1 Bojkova, D. *et al.* Proteomics of SARS-CoV-2-infected host cells reveals therapy targets. *Nature*, doi:10.1038/s41586-020-2332-7 (2020).
