# Supplementary figures and images for: A cross-talk between epithelium and endothelium mediates human alveolar–capillary injury during SARS-CoV-2 infection
Source: Cell Death Dis. 2020 Dec 8;11(12):1042. doi: 10.1038/s41419-020-03252-9 (PMC7721862; doi:10.1038/s41419-020-03252-9)

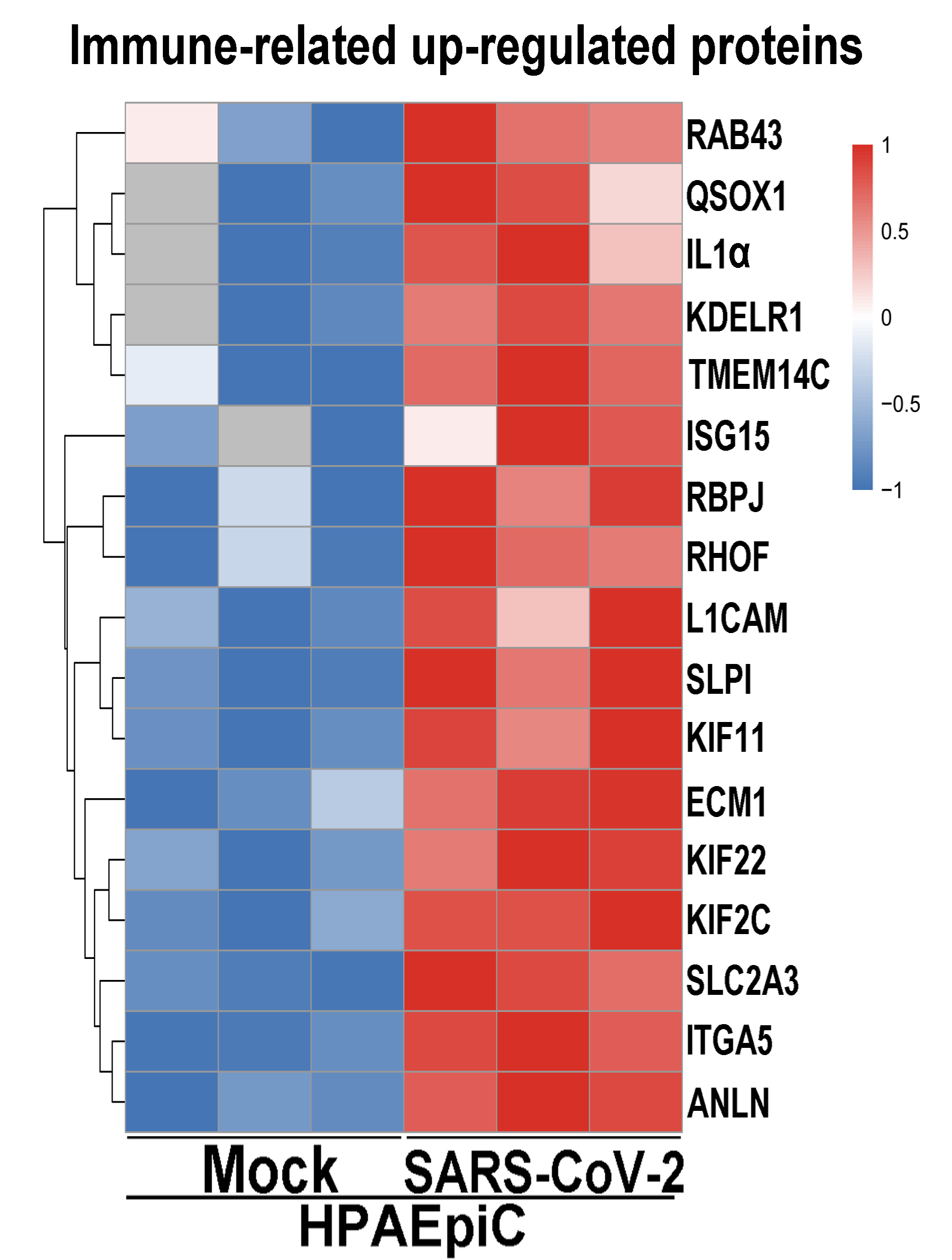

Supplement: Supplementary file 2 — Supplementary Figure 1 [file 41419_2020_3252_MOESM2_ESM.tif]

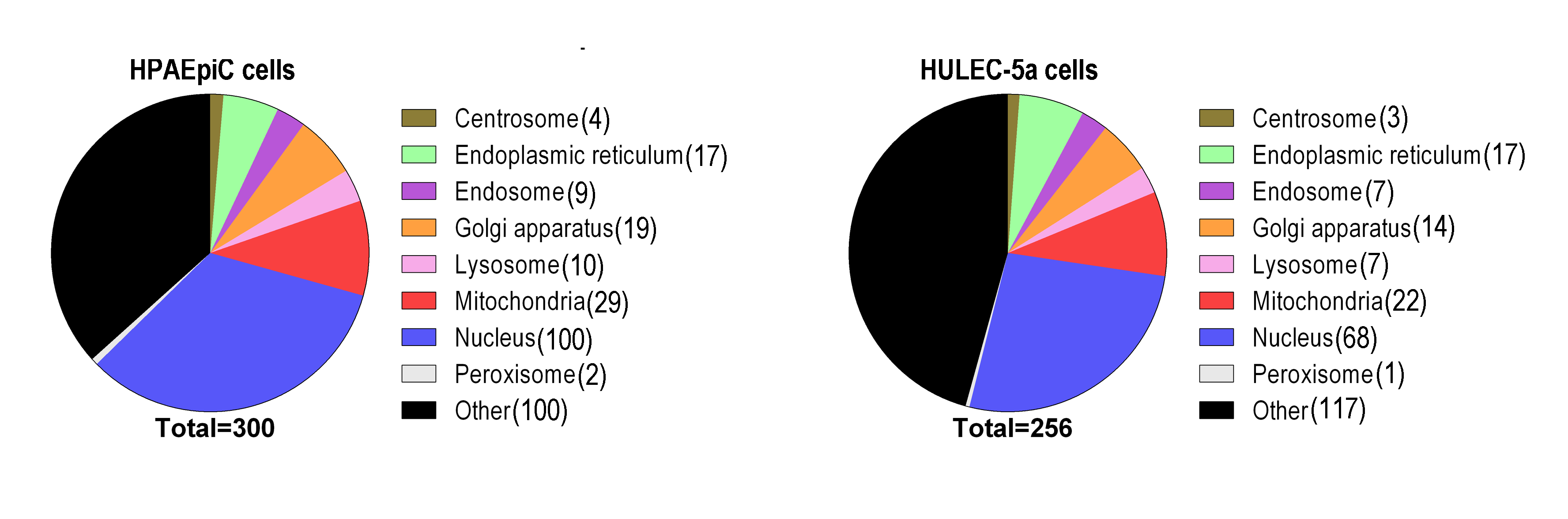

Supplement: Supplementary file 3 — Supplementary Figure 2 [file 41419_2020_3252_MOESM3_ESM.tif]

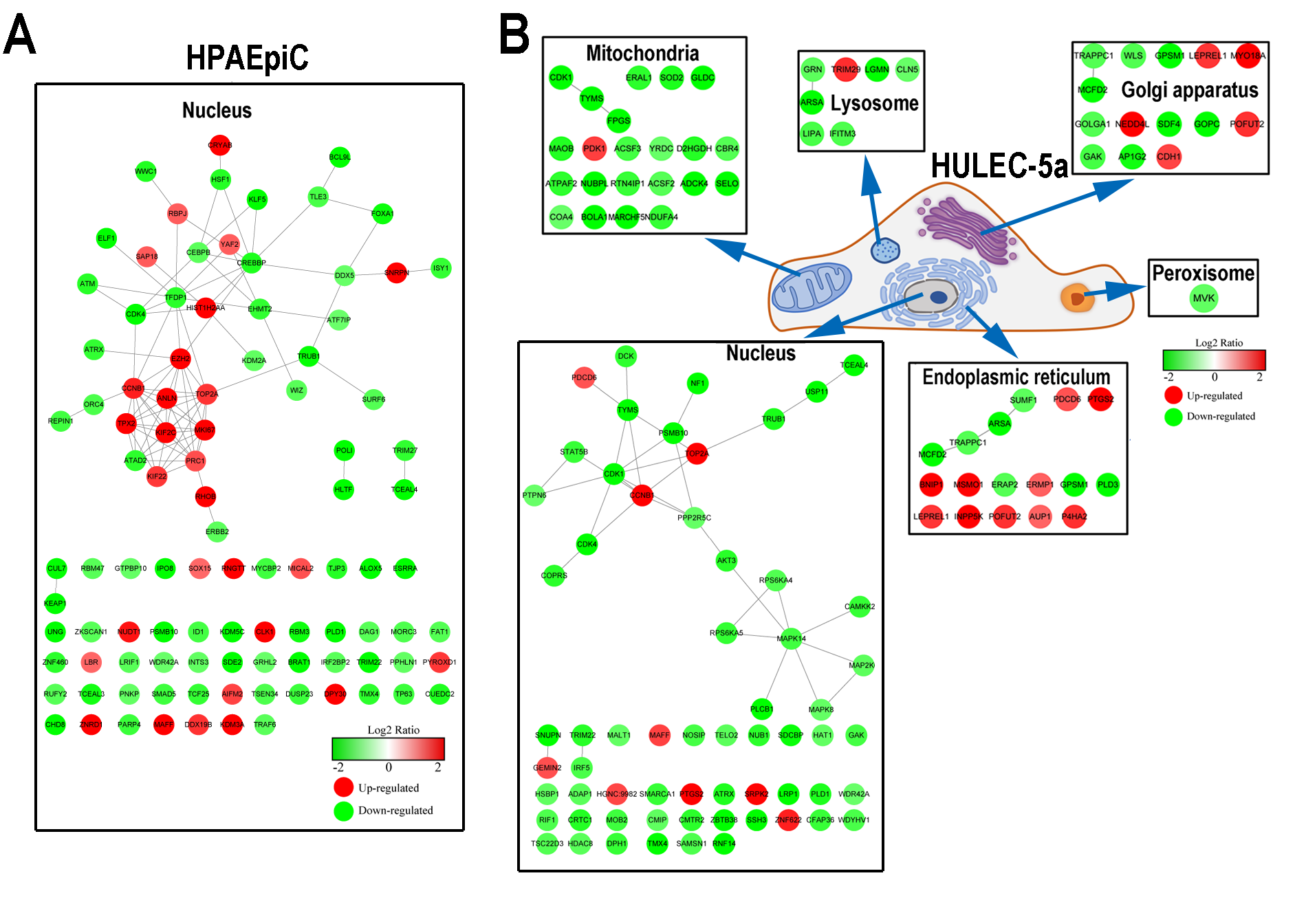

Supplement: Supplementary file 4 — Supplementary Figure 3 [file 41419_2020_3252_MOESM4_ESM.tif]

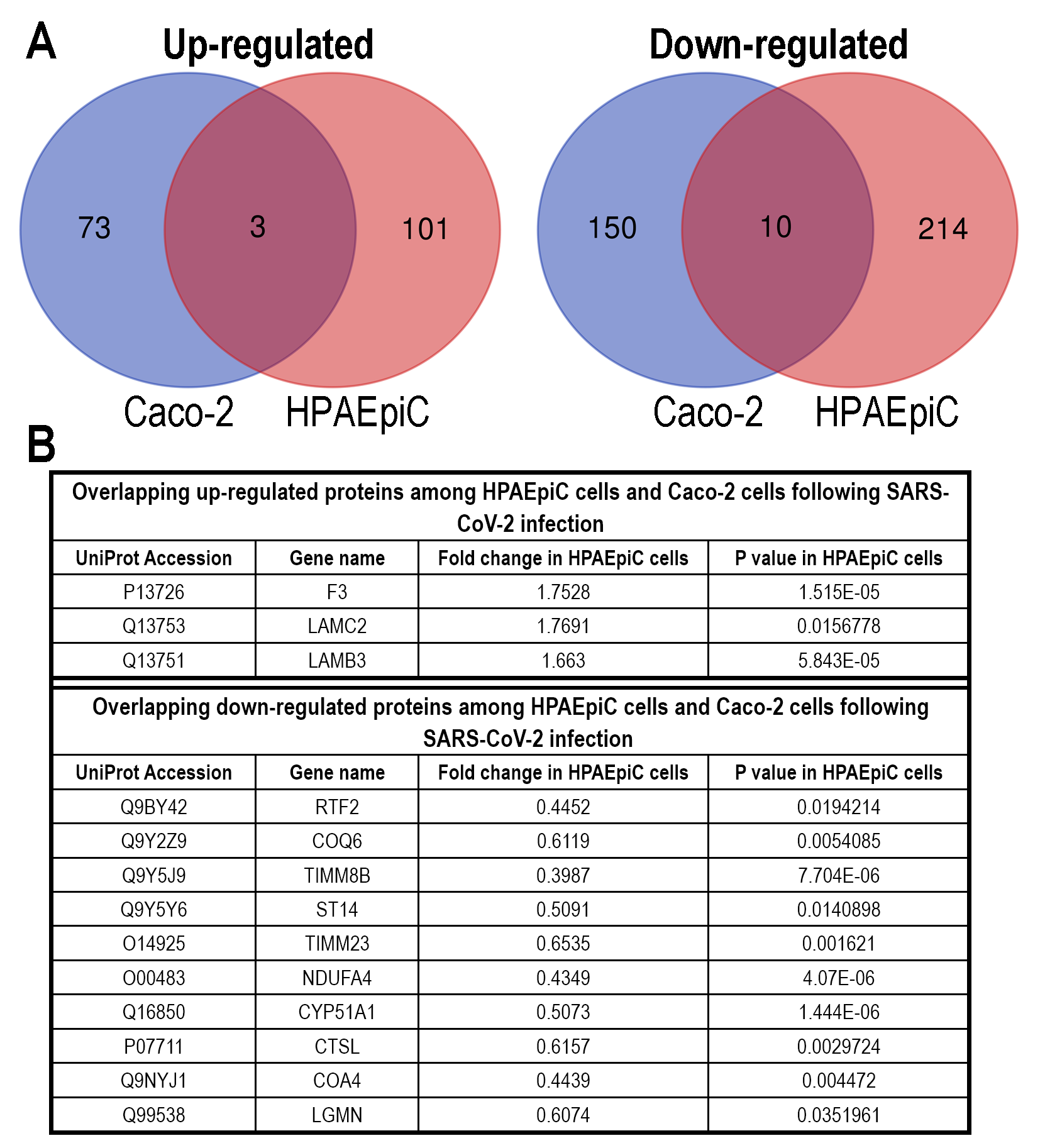

Supplement: Supplementary file 5 — Supplementary Figure 4 [file 41419_2020_3252_MOESM5_ESM.tif]

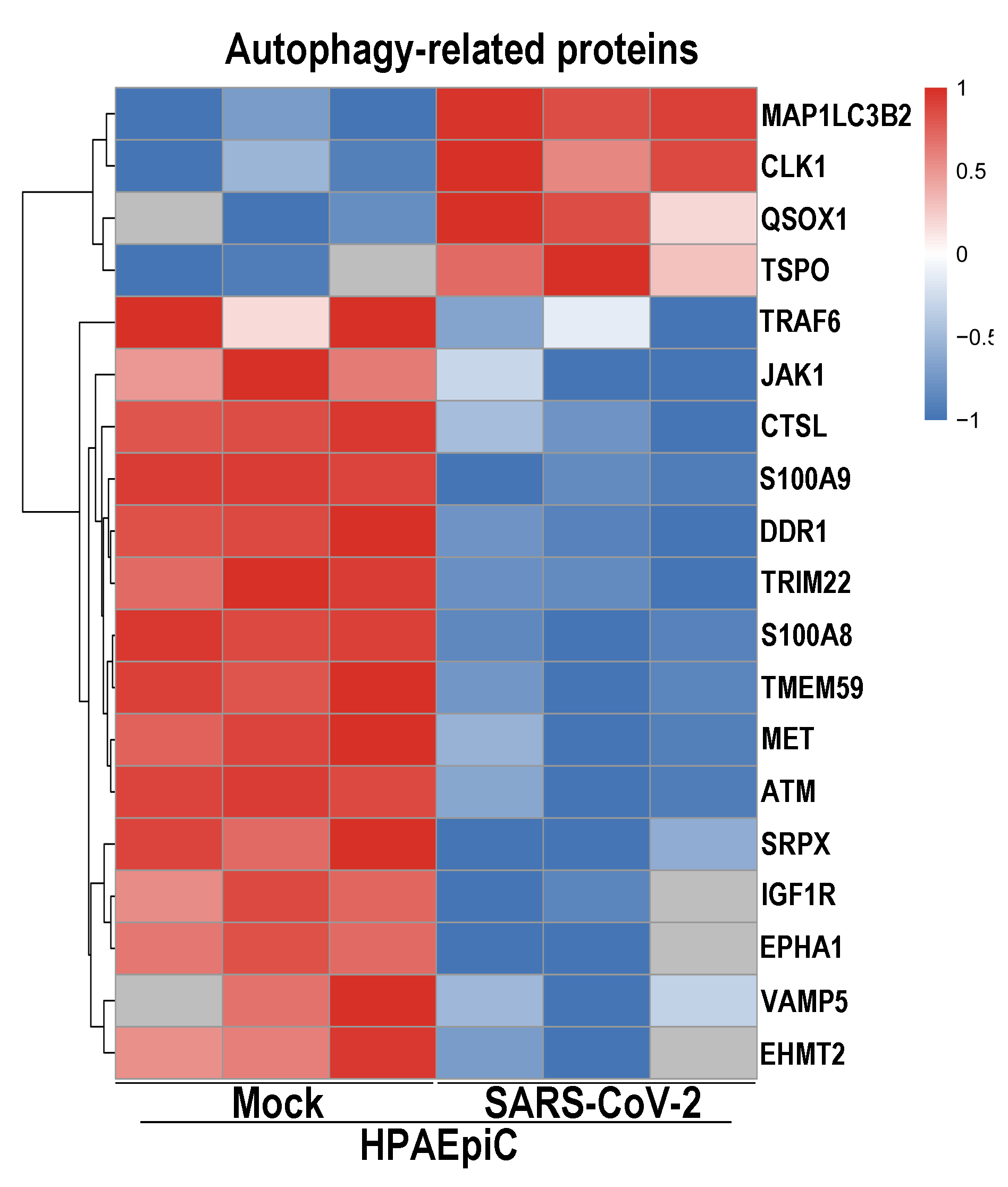

Supplement: Supplementary file 6 — Supplementary Figure 5 [file 41419_2020_3252_MOESM6_ESM.tif]

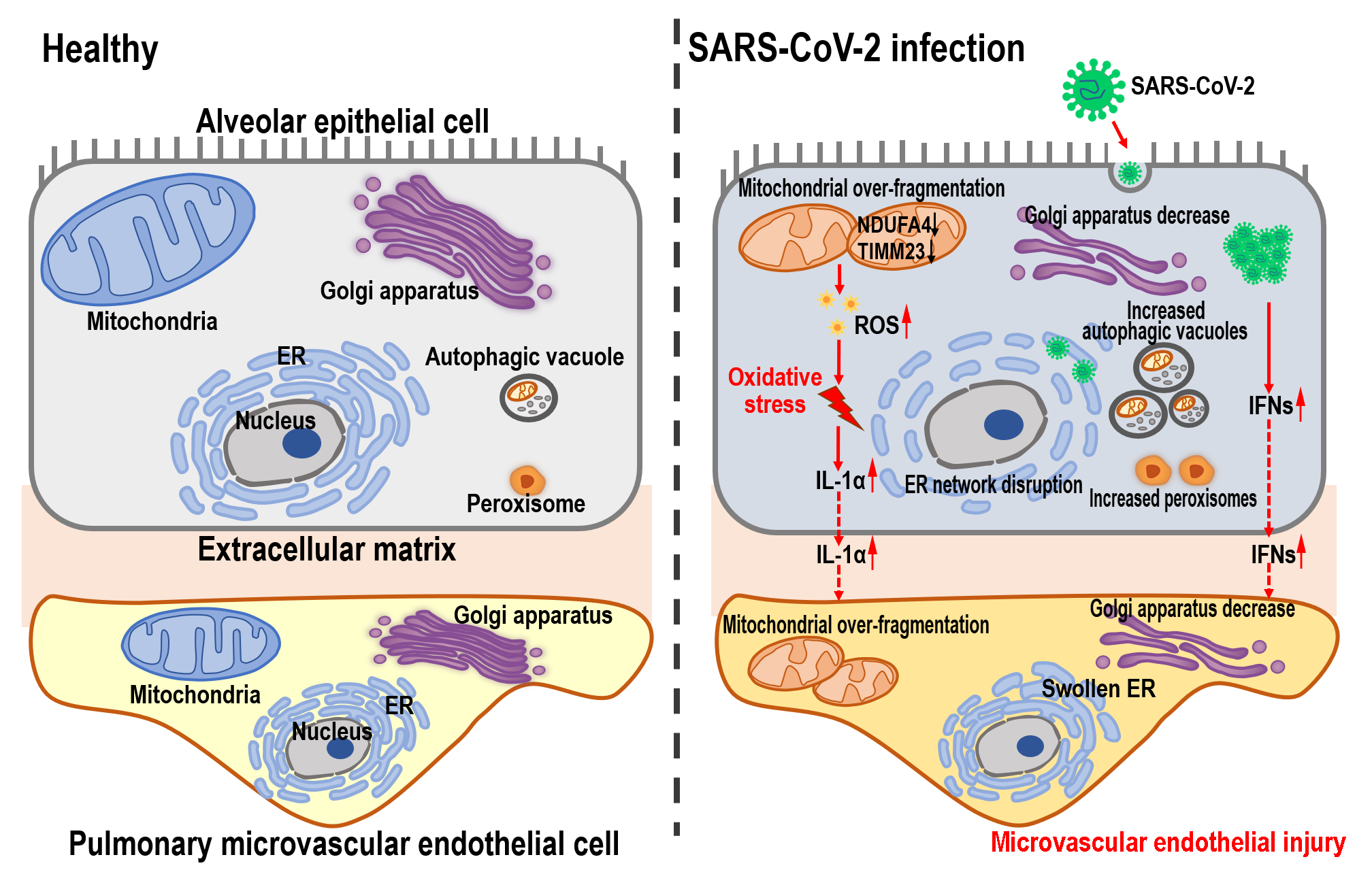

Supplement: Supplementary file 7 — Supplementary Figure 6 [file 41419_2020_3252_MOESM7_ESM.tif]
